# Sympathetic Innervation of Interscapular Brown Adipose Tissue Is Not a Predominant Mediator of Oxytocin-Induced Brown Adipose Tissue Thermogenesis in Female High Fat Diet-Fed Rats

**DOI:** 10.3390/cimb46100679

**Published:** 2024-10-15

**Authors:** Andrew D. Dodson, Adam J. Herbertson, Mackenzie K. Honeycutt, Ron Vered, Jared D. Slattery, Matvey Goldberg, Edison Tsui, Tami Wolden-Hanson, James L. Graham, Tomasz A. Wietecha, Kevin D. O’Brien, Peter J. Havel, Carl L. Sikkema, Elaine R. Peskind, Thomas O. Mundinger, Gerald J. Taborsky, James E. Blevins

**Affiliations:** 1 VA Puget Sound Health Care System, Office of Research and Development Medical Research Service, Department of Veterans Affairs Medical Center, Seattle, WA 98108, USA; anddodson@gmail.com (A.D.D.); adamherb12@gmail.com (A.J.H.); machoney@ucdavis.edu (M.K.H.); rvered@live.com (R.V.); jared.slattery@va.gov (J.D.S.); matveyg@uw.edu (M.G.); edisontsui2000@gmail.com (E.T.); tami.woldenhanson@gmail.com (T.W.-H.); sikkema@uw.edu (C.L.S.); peskind@uw.edu (E.R.P.);; 2 Department of Nutrition, University of California, Davis, CA 95616, USA; jlgraham@ucdavis.edu (J.L.G.); pjhavel@ucdavis.edu (P.J.H.); 3 Division of Metabolism, Endocrinology and Nutrition, Department of Medicine, University of Washington School of Medicine, Seattle, WA 98195, USA; wietechatomasz@gmail.com (T.A.W.); mundin714@gmail.com (T.O.M.); 4 UW Medicine Diabetes Institute, University of Washington School of Medicine, Seattle, WA 98109, USA; cardiac@cardiology.washington.edu; 5 Division of Cardiology, Department of Medicine, University of Washington School of Medicine, Seattle, WA 98195, USA; 6 Department of Molecular Biosciences, School of Veterinary Medicine, University of California, Davis, CA 95616, USA; 7 Department of Psychiatry and Behavioral Sciences, University of Washington School of Medicine, Seattle, WA 98195, USA

**Keywords:** obesity, brown adipose tissue, oxytocin, female rodents

## Abstract

Recent studies have indicated that hindbrain [fourth ventricle (4V)] administration of the neurohypophyseal hormone, oxytocin (OT), reduces body weight, energy intake and stimulates interscapular brown adipose tissue temperature (T_IBAT_) in male diet-induced obese (DIO) rats. What remains unclear is whether chronic hindbrain (4V) OT can impact body weight in female high fat diet-fed (HFD) rodents and whether this involves activation of brown adipose tissue (BAT). We hypothesized that OT-elicited stimulation of sympathetic nervous system (SNS) activation of interscapular brown adipose tissue (IBAT) contributes to its ability to activate BAT and reduce body weight in female high HFD-fed rats. To test this hypothesis, we determined the effect of disrupting SNS activation of IBAT on OT-elicited stimulation of T_IBAT_ and reduction of body weight in DIO rats. We first measured the impact of bilateral surgical SNS denervation to IBAT on the ability of acute 4V OT (0.5, 1, and 5 µg ≈ 0.5, 0.99, and 4.96 nmol) to stimulate T_IBAT_ in female HFD-fed rats. We found that the high dose of 4V OT (5 µg ≈ 4.96 nmol) stimulated T_IBAT_ similarly between sham rats and denervated rats (*p* = NS). We subsequently measured the effect of bilateral surgical denervation of IBAT on the effect of chronic 4V OT (16 nmol/day ≈ 16.1 μg/day) or vehicle infusion to reduce body weight, adiposity and energy intake in female HFD-fed rats (N = 7–8/group). Chronic 4V OT reduced body weight gain (sham: −18.0 ± 4.9 g; denervation: −15.9 ± 3.7 g) and adiposity (sham: −13.9 ± 3.7 g; denervation: −13.6 ± 2.4 g) relative to vehicle treatment (*p* < 0.05) and these effects were similar between groups (*p* = NS). These effects were attributed, in part, to reduced energy intake evident during weeks 2 (*p* < 0.05) and 3 (*p* < 0.05). To test whether these results translate to other female rodent species, we also examined the effect of chronic 4V infusion of OT on body weight and adiposity in two strains of female HFD-fed mice. Similar to what we found in the HFD-fed rat model, we also found that chronic 4V OT (16 nmol/day) infusion resulted in reduced body weight gain, adiposity and energy intake in female DIO C57BL/6J and DBA/2J mice (*p* < 0.05 vs. vehicle). Together, these findings suggest that (1) sympathetic innervation of IBAT is not necessary for OT-elicited increases in BAT thermogenesis and weight loss in female HFD-fed rats and (2) the effects of OT to reduce weight gain and adiposity translate to other female mouse models of diet-induced obesity (DIO).

## 1. Introduction

The neuropeptide, oxytocin (OT), has been largely associated with eliciting prosocial (i.e., pair bonds and increased trust) [1,2] and reproductive behavior (i.e., uterine contraction, milk ejection reflex) [3,4], but the role of OT in the regulation of body weight, particularly in female rodents [5,6,7,8], is not entirely clear. Recent studies have shown that acute intracerebroventricular (ICV) OT administration reduces food intake in female single-minded 1 (SIM1) haploinsufficient mice [9] and female rats [10]. However, few long-term treatment studies exploring the mechanism (s) by which chronic central nervous system (CNS) administration of OT reduces body weight and adiposity in female rodents have been reported.

Although suppression of food intake is thought to contribute, at least in part, to the effects of hindbrain (fourth ventricle (4V)) OT-elicited weight loss in male rodents, the findings from pair-feeding studies from male rodents suggest that OT-elicited reductions of food intake cannot fully explain OT elicited weight loss [11,12,13]. In addition to OT’s well established effects on food intake, previous studies in rodents and nonhuman primates have shown that OT may also evoke weight loss, in part, by stimulating energy expenditure (EE) [14,15,16,17] and lipolysis [12,14,18]. While it is clear that brown adipose tissue thermogenesis (BAT) plays an important role in the regulation of EE (see [19,20] for review), less is known about whether OT’s effects on EE result from (1) non-shivering BAT thermogenesis, (2) spontaneous physical activity-induced thermogenesis [21], (3) non-shivering and shivering thermogenesis in skeletal muscle [22,23] and/or through the anabolic effects of OT on muscle [24,25], (5) white adipose tissue thermogenesis or (6) hormonal mediators (e.g., fibroblast growth factor-21 [26], irisin [27], leptin [28], thyroid hormone [29] or secretin [30,31] (see [32,33] for review). We and others have found that acute injections of OT into either the forebrain (third (3V)) or hindbrain (4V) elevate interscapular BAT temperature (T_IBAT_) (surrogate measure of BAT thermogenesis) [34,35] and/or core temperature [36] in male rats or mice. Furthermore, the onset of OT-elicited weight loss coincides with OT-elicited elevations of T_IBAT_ in male diet-induced obese (DIO) rats [34]. In addition, an earlier study found that chemogenetic excitation of hypothalamic paraventricular nucleus (PVN) OT neurons increases both subcutaneous BAT temperature and EE in *Oxytocin-Ires Cre* mice [37]. In addition, a recent study reported that chronic subcutaneous infusion of OT increases core temperature, IBAT thermogenic gene expression and differentiation of BAT in vitro in male DIO mice [38]. On the other hand, genetic knockdown or pharmacological blockade of OT signaling reduces cold-induced BAT thermogenesis [39,40,41,42], decreases EE [16,17,39,43] and promotes obesity [17,43,44,45] in mice. We recently determined the impact of bilateral surgical sympathetic nervous system (SNS) denervation to IBAT on the ability of chronic hindbrain (4V) OT infusion to reduce body weight and adiposity in male DIO mice. We found that chronic 4V OT produced similar reductions of body weight and adiposity between groups suggesting that SNS innervation of IBAT is not required for OT to reduce body weight and adiposity in male DIO mice [35]. This finding raised the question as to whether OT stimulates IBAT thermogenesis and evokes weight loss through a mechanism that requires increased SNS outflow to IBAT in female high fat diet (HFD)-fed rats and whether the effect of OT on BAT thermogenesis may involve hindbrain oxytocin receptors (OTR). Here, we aimed to determine the role of SNS outflow to IBAT in contributing to the effect of chronic hindbrain (4V) OT to stimulate BAT thermogenesis and evoke weight loss in a female HFD-fed rat model.

Based on our previous findings that linked 4V OT to increases in BAT thermogenesis in male DIO rats, we hypothesized that OT-induced stimulation of SNS outflow to IBAT contributes to its ability to stimulate non-shivering BAT thermogenesis and evoke weight loss in female HFD-fed rats. To assess if SNS innervation of BAT is required for OT to stimulate non-shivering thermogenesis in IBAT (as surrogate measure of energy expenditure), we determined the effects of acute 4V injections of OT (0.5, 1, and 5 μg) on T_IBAT_ in female HFD-fed rats following bilateral surgical SNS denervation to IBAT. To determine whether SNS innervation of IBAT is required for OT to elicit weight loss, we measured the ability of chronic 4V OT (16 nmol/day over 29 days) to decrease body weight and adiposity in female HFD-fed rats following bilateral surgical or sham denervation of IBAT. We subsequently determined if these effects were associated with a reduction of adipocyte size and energy intake. To test whether the effects of chronic 4V OT to reduce body weight and adiposity could translate to other female rodent models of diet-induced obesity (DIO), we also examined the effect of chronic 4V infusion of OT on body weight and adiposity in two different strains of female HFD-fed mice (C57BL/6J and DBA/2J). Our findings suggest that (1) sympathetic innervation of IBAT is not necessary for OT-elicited increases in BAT thermogenesis and weight loss in female HFD-fed rats and (2) the effects of OT to elicit weight loss translate to other mouse models of diet-induced obesity (DIO).

## 2. Materials and Methods

### 2.1. Animals

Adult female Long-Evans rats and C57BL/6J (strain 000664) and DBA/2J (strain 000671) mice were initially obtained from Envigo [Indianapolis, IN (rats)] or [The Jackson Laboratory; Bar Harbor, ME (mice)] and maintained for at least 4 months on a HFD prior to study onset. All animals were housed individually in Plexiglas cages in a temperature-controlled room (22 ± 2 °C) under a 12:12-h light-dark cycle. All rats and mice were maintained on a 6 a.m. (lights on)/6 p.m. (lights off) light cycle. Rats and mice had *ad libitum* access to water and a HFD providing 60% kcal from fat [Research Diets, D12492 (rats) or D12492i (mice), New Brunswick, NJ, USA]. The research protocols were approved both by the Institutional Animal Care and Use Committee of the Veterans Affairs Puget Sound Health Care System (VAPSHCS) and the University of Washington in accordance with NIH Guidelines for the Care and Use of Animals.

### 2.2. Drug Preparation

The beta-3 adrenergic receptor (β3-AR) agonist**,** CL 316243 (Tocris/Bio-Techne Corporation, Minneapolis, MN)**,** was solubilized in sterile water on each day of each experiment (Study 1). OT acetate salt (Bachem Americas, Inc., Torrance, CA, USA) was solubilized in sterile water on each day of each study (Study 2). For Studies, 3–5, OT acetate salt (Bachem Americas, Inc., Torrance, CA, USA) was dissolved in sterile water and subsequently added to Alzet^®^ minipumps (model 2004; DURECT Corporation, Cupertino, CA, USA) and primed in sterile vehicle (0.9% saline) at 37 °C for approximately 40 h.

### 2.3. SNS Denervation Procedure

The procedure for SNS denervation of IBAT has been described previously [35]. Briefly, a dissecting microscope (Leica M60/M80; Leica Microsystems, Buffalo Grove, IL, USA) was used for the denervation/sham surgeries. Rats were treated pre-operatively with the analgesic ketoprofen (2 mg/kg; Fort Dodge Animal Health, Overland Park, KS, USA) prior to the completion of the denervation or sham procedure. This IBAT denervation procedure was combined with transponder implantations for studies that involved IBAT temperature measurements in response to acute (Studies 1–2) IP or 4V administration. Animals were allowed to recover for approximately 5–7 days prior to implantation of 4V cannulas.

### 2.4. 4V Cannulations for Acute Injections in Rats

The procedure for 4V cannulations for acute injections in rats has been described previously [34,46]. Briefly, rats were implanted with a cannula (P1 Technologies, Roanoke, VA, USA) that was directed towards the 4V [47,48,49]. Rats were initially anesthetized with isoflurane and subsequently positioned on a stereotaxic device [Digital Lab Standard Stereotaxic, Rat, (Item 51900), Stoelting Co., Wood Dale, IL, USA] with the incisor bar fixed at 3.3 mm below the interaural line. A 26-gauge cannula (P1 Technologies) was stereotaxically implanted into the 4V [−3.5 mm caudal to the interaural line; 1.4 mm lateral to the midline, and 6.2 mm ventral to the skull surface [50] and fastened to the surface of the skull with dental acrylic and stainless-steel screws. Rats were treated with the analgesic ketoprofen (2 mg/kg; Fort Dodge Animal Health) and the antibiotic enrofloxacin (5 mg/kg; Bayer Healthcare LLC., Animal Health Division, Shawnee Mission, KS, USA) at the completion of the 4V cannulations. Animals were allowed to recover for at least 10 days prior to study onset.

### 2.5. 4V Cannulations for Chronic Infusions in Rats

The procedure for 4V cannulations for chronic infusions in rats has been described previously [34,46]. Briefly, rats were implanted with a cannula within the 4V with a side port that was connected to an osmotic minipump (model 2004, DURECT Corporation) [34,46,51]. Rats were initially anesthetized with isoflurane anesthesia and subsequently placed in a stereotaxic device [Digital Lab Standard Stereotaxic, Rat, (Item 51900), Stoelting Co.] with the incisor bar fixed at 3.3 mm below the interaural line. A 30-gauge cannula (P1 Technologies) was stereotaxically directed into the 4V [−3.5 mm caudal to the interaural line; 1.4 mm lateral to the midline, and 7.2 mm ventral to the skull surface [50]] and secured to the surface of the skull with stainless steel screws and dental acrylic. Rats were given the analgesic ketoprofen (2 mg/kg; Fort Dodge Animal Health) and the antibiotic enrofloxacin (5 mg/kg; Bayer Healthcare LLC., Animal Health Division, Shawnee Mission, KS, USA) at the completion of the 4V cannulations and were allowed to recover at least 10 days prior to implantation of osmotic minipumps.

### 2.6. 4V Cannulations for Chronic Infusions in Female C57BL/6J and DBA/2J Mice

The procedure for 4V cannulations for chronic infusions in mice has been described previously [52]. Mice were implanted with a cannula within the 4V with a side port that was connected to an osmotic minipump (model 2004, DURECT Corporation) as previously described [52]. Mice were initially anesthetized with isoflurane anesthesia and subsequently positioned on a stereotaxic device [Digital Just for Mouse Stereotaxic, (Item 51730D), Stoelting Co.] with the incisor bar positioned 4.5 mm below the interaural line. A 30-gauge cannula (P1 Technologies) was stereotaxically positioned into the 4V of either female C57BL/6J or DBA/2J mice (−5.9 mm caudal to bregma; 0.4 mm lateral to the midline, and 3.7 mm ventral to the skull surface) [53] and secured to the surface of the skull with dental cement and stainless steel screws. Mice were treated with the analgesic ketoprofen (5 mg/kg; Fort Dodge Animal Health) and the antibiotic enrofloxacin (5 mg/kg; Bayer Healthcare LLC., Animal Health Division Shawnee Mission, KS, USA) at the completion of the 4V cannulations and were allowed to recover for at least 10 days prior to implantation of osmotic minipumps.

### 2.7. Implantation of Temperature Transponders Underneath IBAT

The procedure for temperature transponder implantations underneath IBAT in rats and mice has been described previously [35,52,54]. Animals were initially anesthetized with isoflurane and had the dorsal surface along the upper midline of the back shaved and the area was scrubbed with 70% ethanol followed by betadine swabs. A one-inch incision was made at the midline of the interscapular area. The temperature transponder (14 mm long/2 mm wide) (HTEC IPTT-300; Bio Medic Data Systems, Inc., Seaford, DE, USA) was implanted underneath the left IBAT pad as previously described [34,35,52,54,55,56] and secured in place by suturing it to the brown fat pad with sterile silk suture. The interscapular incision was closed with Nylon sutures (5-0), which were removed in awake animals 10–14 days after surgery. Rats or mice were treated pre-operatively with the analgesic ketoprofen [rats: 2 mg/kg; mice: 5 mg/kg (Fort Dodge Animal Health)] prior to the completion of the temperature transponder implantation procedure.

### 2.8. Acute IP or 4V Injections and Measurements of T_IBAT_

On an experimental day, 4-h fasted animals received either IP (CL 316243 or sterile water vehicle; 0.1 mL/kg injection volume) or 4V injections (OT or saline vehicle; 1 μL injection volume) during the early part of the light cycle. Injections were completed in a crossover design over approximately 7-day (CL 316243) or 48-h (OT) intervals such that each animal served as its own control. Animals remained fasted for an additional 4 h (Study 3–4) during the course of the T_IBAT_ measurements. A handheld reader (DAS-8007-IUS Reader System; Bio Medic Data Systems, Inc.) was used to collect measurements of T_IBAT_. Rats underwent all treatments in a randomized order separated by at least 48-h (OT) or 7–8 days (CL 316243) between treatments.

### 2.9. Body Composition

The procedure for measuring body composition in mice and rats has been described previously [35,54]. Briefly, determinations of lean body mass and fat mass were made on un-anesthetized mice and rats by quantitative magnetic resonance using an EchoMRI 4-in-1-700^TM^ instrument (Echo Medical Systems, Houston, TX, USA) at the VAPSHCS Rodent Metabolic Phenotyping Core. Measurements were taken prior to 4V cannulations and/or minipump implantations as well as at the end of the infusion period.

### 2.10. Tissue Collection for Norepinephrine (NE) Content Measurements

Rats were euthanized by rapid conscious decapitation at 8 weeks (Study 1–2) or 7–12 weeks (Study 3) post-sham or denervation procedure. Trunk blood and tissues (IBAT, EWAT, IWAT, liver and/or pancreas) were collected from 4-h fasted rats. Tissue was rapidly removed, wrapped in foil and frozen in liquid N2. Samples were stored frozen at −80 °C until analysis. Note that rapid conscious decapitation was used in place of anesthesia when collecting tissue for NE content as anesthesia can cause the release of NE from SNS terminals within the tissue [57].

### 2.11. NE Content Measurements (Biochemical Confirmation of IBAT Denervation Procedure)

The procedure for measuring NE content has been described previously [58]. Specifically, NE content was measured in IBAT, EWAT, IWAT, liver and/or pancreas using previously established techniques [35,58]. Successful denervation was noted by ≥60% reduction in IBAT NE content as previously noted [35,59]. Experimental animals that did not meet this criterion were excluded from the data analysis.

### 2.12. Study Protocols

#### 2.12.1. Study 1: Determine if Surgical Denervation of IBAT Changes the Ability of the β-3R Agonist, CL 316243, to Increase T_IBAT_ in DIO Rats

Rats (N = 13 at study onset) from Study 2 were used in these studies. Rats were fed *ad libitum* and maintained on HFD for approximately 4.5 months prior to underdoing sham or SNS denervation procedures and implantation of temperature transponders underneath the left IBAT depot. Animals were subsequently implanted with 4V cannulas approximately 1 week following sham/denervation procedures and implantation of temperature transponders. Rats were allowed to recover for at least 2 weeks during which time they were adapted to a daily 4-h fast, handling and mock injections. On an experimental day, 4-h fasted rats received Cl 316243 (0.1 or 1 mg/kg) or vehicle (sterile water) during the early part of the light cycle in a crossover design at approximately 7-day intervals such that each animal served as its own control (approximately 1–3 weeks post-sham or denervation procedures). T_IBAT_ was measured at baseline (−2 h; 9:00 a.m.), immediately prior to IP injections (0 h; 9:45–10:00 a.m.), and at 0.25, 0.5, 0.75, 1, 1.25, 1.5, 2, 3, 4, and 24-h post-injection (10:00 a.m.). Food intake and body weight were measured daily. Daily food intake was determined by measuring the difference in weight of the high fat diet pre- vs. post-intervention of a 24-h period and converting grams/day to units of energy intake/day (kcal/day; 5.24 kcal/gram [60]). This dose range was based on doses of CL 316243 found to be effective at reducing food intake and weight gain in rats [52,61]. Animals were euthanized by rapid conscious decapitation at 13 weeks post-sham or denervation procedure.

#### 2.12.2. Study 2: Determine the Extent to Which OT-Induced Activation of Sympathetic Outflow to IBAT Contributes to Its Ability to Increase T_IBAT_ in DIO Rats

Rats (N = 16 at study onset) from Study 1 were used in these studies. On an experimental day, 4-h fasted rats received OT (1 or 5 μg/μL) or vehicle during the early part of the light cycle in order to maximize the effects of OT [17,52] during a time when circulating NE levels [62] and IBAT catecholamine levels are lower [63]. Injections were completed in a crossover design at approximately 48-h to 72-h intervals such that each animal served as its own control (approximately 4-weeks post-sham or denervation procedures). T_IBAT_ was measured at baseline (−2 h; 9:00 a.m.), immediately prior to 4V injections (0 h; 9:45–10:00 a.m.), and at 0.25, 0.5, 0.75, 1, 1.25, 1.5, 2, 3, 4, and 24-h post-injection (10:00 a.m.). Food intake and body weight were measured daily. This dose range was based on doses of 4V OT found to be effective at stimulating T_IBAT_ in male DIO rats in previous studies [34].

In addition, we examined the impact of a lower dose of OT (0.5 μg/μL) in an identical manner following the completion of the initial studies in Study 2.

#### 2.12.3. Study 3A: Determine the Extent to Which OT-Induced Activation of Sympathetic Outflow to IBAT Contributes to Its Ability to Reduce Weight Gain in Female HFD-Fed Rats

Rats (N = 30 at study onset) were used for these studies. Animals were fed *ad libitum* and maintained on HFD for approximately 5.25 months prior to receiving implantations of temperature transponders underneath IBAT, 4V cannulas and subcutaneous minipumps to infuse vehicle or OT (16 nmol/day) over 29 days as previously described [34]. This dose was selected based on a dose of 4V OT found to be effective at reducing body weight in male DIO rats [34]. Daily food intake and body weight were also tracked for 29 days. Animals were euthanized by rapid conscious decapitation at 7 weeks post-sham or denervation procedure. Trunk blood and tissues [IBAT, epididymal white adipose tissue (EWAT), inguinal white adipose tissue (IWAT), liver and pancreas)] were collected from 4-h fasted rats and tissues were subsequently analyzed for IBAT NE content to confirm success of denervation procedure relative to sham operated animals and other tissues (EWAT, IWAT, liver and pancreas).

#### 2.12.4. Study 3B: Determine the Extent to Which 4V OT Impacts Thermogenic Gene Expression in IBAT and IWAT in Female HFD-Fed Rats

Rats from Study 3A were used for these studies. All rats received chronic infusions of 4V vehicle or OT (16 nmol/day) and were euthanized by rapid conscious decapitation following a 4-h fast.

#### 2.12.5. Study 4A: Determine the Effects of Chronic 4V OT Treatment on Body Weight, Adiposity and Energy Intake in Female HFD-Fed C57BL/6J Mice

Female mice (N = 20 at study onset) were fed *ad libitum* and maintained on HFD for approximately 4.5 months prior to being implanted with temperature transponders underneath IBAT. Mice were allowed up to 1-week post-op recovery prior to receiving 4V cannulas. Mice were allowed up to 2 weeks post-op recovery prior to being implanted with minipumps as previously described [34]. T_IBAT_ was measured daily at baseline (−2 h; 9:00 a.m.) and immediately prior to access to food (10:00 a.m.). Daily food intake, body weight and T_IBAT_ were tracked for 28 days.

#### 2.12.6. Study 4B: Determine the Effects of Chronic 4V OT Treatment on Body Weight, Adiposity and Energy Intake in Female DIO DBA2J Mice

Female mice (N = 20 at study onset) were used for these studies. Animals were fed *ad libitum* and maintained on HFD for approximately 4.5 months prior to being implanted with temperature transponders underneath IBAT. Mice were allowed up to 2 weeks post-op recovery prior to receiving 4V cannulas. Mice were allowed up to 4 weeks post-op recovery prior to being implanted with minipumps as previously described [34]. T_IBAT_ was measured daily at baseline (−2 h; 9:00 a.m.) and immediately prior to access to food (10:00 a.m.). Daily food intake, body weight and T_IBAT_ were tracked for 28 days.

#### 2.12.7. Study 5: Determine the Effects of Chronic Systemic OT Treatment (16 and 50 nmol/day) on Body Weight, Adiposity and Energy Intake in Female DIO DBA/2J Mice

Female mice (N = 22 mice at study onset) were fed *ad libitum* and maintained on HFD for approximately 4.5 months prior to being implanted with temperature transponders underneath IBAT. Mice were allowed up to 4 weeks post-op recovery prior to being implanted with subcutaneous (SC) minipumps as previously described [34]. T_IBAT_ was measured daily at baseline (−2 h; 9:00 a.m.) and immediately prior to access to food (10:00 a.m.). Daily food intake, body weight and T_IBAT_ were tracked for 27 days.

### 2.13. Blood Collection

Trunk blood (Study 1–2) or blood from cardiac stick (Study 3–5) was collected from 4-h fasted rats or mice within a 2-h window towards the beginning of the light cycle (10:00 a.m.–12:00 p.m.) as previously described in DIO CD^®^IGS and Long-Evans rats and C57BL/6J mice [34,48]. Treatment groups were counterbalanced at time of euthanasia to avoid time of day bias. Blood samples [up to 1 mL (mice) or 3 mL (rats)] were collected from trunk or via cardiac puncture in chilled K2 EDTA Microtainer Tubes (Becton-Dickinson, Franklin Lakes, NJ, USA). Whole blood was centrifuged at 6000 rpm for 1.5-min at 4 °C; plasma was removed, aliquoted and stored at −80 °C for subsequent analysis.

### 2.14. Plasma Hormone Measurements

Plasma leptin and insulin were measured using electrochemiluminescence detection [Meso Scale Discovery (MSD^®^), Rockville, MD, USA] using established procedures [34,64]. Intra-assay coefficient of variation (CV) for leptin was 2.8% and 2.4% for rat and mouse, respectively. Intra-assay CV for insulin was 2.7% and 2.4% for rat and mouse, respectively. The range of detectability for the leptin assay is 0.07–51.9 ng/mL and 0.069–50 ng/mL for insulin. Plasma glucagon (Mercodia, Winston Salem, NC, USA), fibroblast growth factor-21 (FGF-21) (R&D Systems, Minneapolis, MN, USA) and irisin (AdipoGen, San Diego, CA, USA) levels were determined by ELISA. The intra-assay CV for glucagon was 1.6% and 1.9% for rat and mouse, respectively, and the range of detection was 2–182 pmol/L. The intra-assay CV for FGF-21 was 2.7% and 2.3% for rat and mouse, respectively. The intra-assay CV for irisin was 6.9% for mouse (not obtained for rat). The ranges of detectability were 31.3–2000 pg/mL (FGF-21) and 0.078–5 μg/mL (irisin). Plasma adiponectin was also measured using ELISA (Alpco, Salem, NH, USA) using established procedures [34,64]. Intra-assay CV for adiponectin was 1.7% and 1.6% for rat and mouse, respectively. The range of detectability for the adiponectin assay is 0.25–10 ng/mL (rat) and 0.025–1 ng/mL (mice). The data were normalized to historical values using a pooled plasma quality control sample that was assayed in each plate.

### 2.15. Blood Glucose and Lipid Measurements

Blood was collected for glucose measurements by tail vein nick in 4-h fasted rats and measured with a glucometer using the AlphaTRAK 2 blood glucose monitoring system (Abbott Laboratories, Abbott Park, IL, USA) [34,65]. Total cholesterol (TC) [Fisher Diagnostics (Middletown, VA, USA)] and free fatty acids (FFAs) [Wako Chemicals USA, Inc., Richmond, VA, USA)] were measured using an enzymatic-based kits. Intra-assay CVs for TC were 3.5% and 3.4% for rat and mouse, respectively. Intra-assay CV for FFA were 1.4% and 2.5% for rat and mouse, respectively. These assay procedures have been validated for rodents [66].

### 2.16. Adipose Tissue Processing for Adipocyte Size

Adipose tissue depots were collected at the end of the infusion period in DIO rats from Study 3B (EWAT). EWAT was processed as previously described [35,46,52,54]. EWAT was dissected and placed in 4% paraformaldehyde-PBS for 24 h and then placed in 70% ethanol (EtOH) prior to paraffin embedding. Sections (5 μm) sampled were obtained using a rotary microtome, slide-mounted using a floatation water bath (37 °C), and baked for 30 min at 60 °C to give approximately 15–16 slides/fat depot with two sections/slide.

### 2.17. Adipocyte Size Analysis

Adipocyte size analysis was performed as previously described [35,46,52,54]. Analysis was completed on deparaffinized and digitized EWAT sections. The average cell area from two randomized photomicrographs was determined using the built-in particle counting method of ImageJ software (https://imagej.net/ij/, assessed date 30 September 2024) (National Institutes of Health, Bethesda, MD, USA). Slides were visualized using bright field on an Olympus BX51 microscope (Olympus Corporation of the Americas; Center Valley, PA, USA) and photographed using a Canon EOS 5D SR DSLR (Canon U.S.A., Inc., Melville, NY, USA) camera at ×10 magnification. Values for each tissue within a treatment were averaged to obtain the mean of the treatment group.

### 2.18. Tissue Collection for Quantitative Real-Time PCR (qPCR)

Tissue (IBAT and IWAT) was collected from a subset of 4-h (Study 3B). IBAT and IWAT were collected within a 2-h window towards the start of the light cycle (10:00 a.m.–12:00 p.m.) as previously described in DIO CD^®^IGS/Long-Evans rats and C57BL/6J mice [34,48,52]. Tissue was rapidly removed, wrapped in foil and frozen in liquid N2. Samples were stored frozen at −80 °C until analysis.

### 2.19. qPCR

RNA extracted from samples of IBAT and IWAT (Study 3B) were analyzed using the RNeasy Lipid Mini Kit (Qiagen Sciences Inc., Germantown, MD, USA) followed by reverse transcription into cDNA using a high-capacity cDNA archive kit (Applied Biosystems, Foster City, CA, USA). Quantitative analysis for relative levels of mRNA in the RNA extracts was measured in duplicate by qPCR on an Applied Biosystems 7500 Real-Time PCR system (Thermo Fisher Scientific, Waltham, MA, USA) and normalized to the cycle threshold value of Nono mRNA in each sample. The TaqMan^®^ probes used in the study were Thermo Fisher Scientific Gene Expression Assay probes. The probe for rat *Nono* (Rn01418995_g1), uncoupling protein-1 (UCP-1) (*Ucp1*; catalog no. Rn00562126_m1), β1-adrenergic receptor (β1-AR) (*Adrb1*; catalog no. Rn00824536_s1), β3-adrenergic receptor (β3-AR) (*Adrb3*; catalog no. Rn01478698_g1), type 2 deiodinase (D2) (*Dio2*; catalog no. Rn00581867_m1), PR domain containing 16 (*Prdm16*; catalog no. Rn01516224_m1), G-protein coupled receptor 120 (*Gpr120*; catalog no. Rn01759772_m1), cell death-inducing DNA fragmentation factor alpha-like effector A (*Cidea*; catalog no. Rn04181355_m1), and peroxisome proliferator-activated receptor gamma coactivator 1 alpha (*Ppargc1a*; catalog no. Rn00580241_m1) were acquired from Thermo Fisher Scientific. Relative amounts of target mRNA were determined using the Comparative C_T_ or 2^−ΔΔCT^ method [67] following adjustment for the housekeeping gene, Nono. Specific mRNA levels of all genes of interest were normalized to the cycle threshold value of *Nono* mRNA in each sample and expressed as changes normalized to controls (vehicle/sham treatment).

### 2.20. Statistical Analyses

All results are expressed as means ± SE. Comparisons between multiple groups involving between-subjects designs were made using one-way ANOVA as appropriate, followed by a post-hoc Fisher’s least significant difference test. Comparisons involving within-subjects designs were made using a one-way repeated-measures ANOVA followed by a post-hoc Fisher’s least significant difference test. Analyses were performed using the statistical program SYSTAT (https://grafiti.com/systat/, accessed date 30 September 2024) (Systat Software, Point Richmond, CA, USA). Differences were considered significant at *p* < 0.05, 2-tailed. Non-statistical trends (0.05 < *p* < 0.1) have been included in the analysis where appropriate.

## 3. Results

### 3.1. Study 1: Determine if Surgical Denervation of IBAT Changes the Ability of the β3-AR Agonist, CL 316243, to Increase T_IBAT_ in Female HFD-Fed Rats

Our objective was to extend recently published results in a mouse model [35] and verify there was no functional impairment in the ability of IBAT to respond to direct β3-AR stimulation as a result of the denervation procedure relative to female sham operated rats. As expected, female HFD-fed rats were borderline obese as confirmed by both body weight (336.8 ± 7.8 g) and adiposity (99.0 ± 6.7 g fat mass; 28.9 ± 1.3% body fat) after being maintained on the HFD for approximately 4 months prior undergoing sham/IBAT denervation surgeries.

All IBAT tissues from Study 1/Study 2 animals were analyzed for IBAT NE content and only 1 out of 5 animals was removed on account of having a failed IBAT denervation procedure. Surgical IBAT denervation resulted in a 76.9 ± 2.7% reduction of IBAT NE content relative to sham-operated control rats [(F(1,10) = 18.975, *p* = 0.001). Similar to what we reported in a mouse model [35], NE content was unaltered in IWAT, EWAT, liver or pancreas in IBAT denervated rats relative to sham-operated rats (*p* = NS). As expected [35], there was no significant difference in body weight between female sham-operated and IBAT denervated rats at the conclusion of the study (*p* = NS).

**Figure 1:**
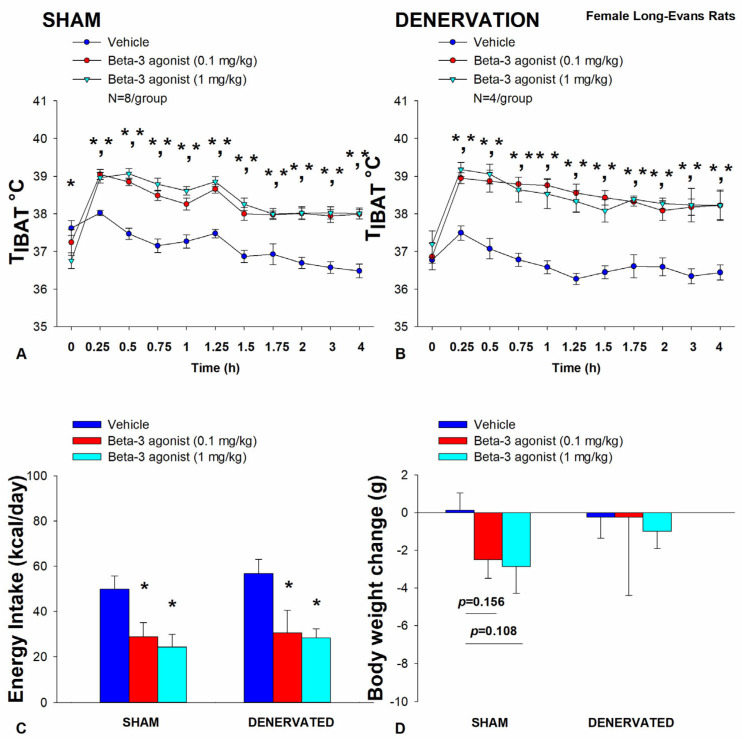
(**A**–**D**) **Effect of systemic β3-AR agonist (CL 316243) administration (0.1 and 1 mg/kg) on IBAT temperature (T_IBAT_), energy intake and body weight post-sham or IBAT denervation in female HFD-fed rats.** Rats were maintained on HFD (60% kcal from fat; N = 4–8/group) for approximately 4.5 months prior to undergoing a sham or bilateral surgical IBAT denervation and implantation of temperature transponders underneath IBAT. Animals were subsequently adapted to a 4-h fast prior to receiving IP injections of CL 316243 (0.1 or 1 mg/kg, IP) or vehicle (sterile water) where each animal received each treatment at approximately 7-day intervals. (**A**,**B**) Effect of CL 316243 on T_IBAT_ in (**A**) sham operated or (**B**) IBAT denervated DIO rats; (**C**) Effect of CL 316243 on change in energy intake in sham or IBAT denervated DIO rats; (**D**) Effect of CL 316243 on change in body weight in sham or IBAT denervated DIO rats. Data are expressed as mean ± SEM. * *p* < 0.05 CL 316243 vs. vehicle.

In sham-operated rats, CL 316243 (1 mg/kg) stimulated T_IBAT_ throughout the post-injection measurement period (0.25, 0.5, 0.75, 1, 1.25, 1.5, 1.75, 2, 3 and 4-h post-injection). In addition, the lower dose of CL 316243 (0.1 mg/kg) also elevated T_IBAT_ throughout the post-injection measurement period (0.25, 0.5, 0.75, 1, 1.25, 1.5, 1.75, 2, 3 and 4-h post-injection) (*p* < 0.05; Figure 1A).

Likewise, in IBAT denervated animals, CL 316243 (1 mg/kg) also stimulated T_IBAT_ at 0.25, 0.5, 0.75, 1, 1.25, 1.5, 1.75, 2, 3 and 4-h post-injection. CL 316243 (0.1 mg/kg) also stimulated T_IBAT_ at 0.25, 0.5, 0.75, 1, 1.25, 1.5, 1.75, 2, 3 and 4-h post-injection (*p* < 0.05; Figure 1B).

Notably, there was no significant difference in the ability of CL 316243 to (0.1 or 1 mg/kg) stimulate T_IBAT_ when averaged over the 1-h or 4-h post-injection period between sham and IBAT denervated animals (*p* = NS).

Collectively, these findings indicate that IBAT denervation did not result in a functional change in the effectiveness of CL 316243 to increase BAT thermogenesis relative to sham-operated animals.

#### 3.1.1. Energy Intake

In sham-operated rats, CL 316243 decreased daily energy intake at both the low (0.1 mg/kg) and high dose (1 mg/kg) by 42.3 and 51.4% (*p* < 0.05). Likewise, in IBAT denervated animals, CL 316243 also decreased daily energy intake at both the low (0.1 mg/kg) and high (1 mg/kg) doses (*p* < 0.05) by 46.2 and 50.4%, respectively (Figure 1C).

#### 3.1.2. Body Weight

CL 316243 had no effect on either body weight or body weight gain in either sham or IBAT denervated rats (*p* = NS; Figure 1D). CL 316243 tended to reduce body weight gain at the high dose (1 mg/kg) in the sham-operated group but this did not reach significance (*p* = 0.111).

Similar to what we observed on T_IBAT_, there was no significant difference in the ability of CL 316243 (0.1 or 1 mg/kg) to reduce energy intake between sham and IBAT denervated rats (*p* = NS).

Collectively, these results indicate that IBAT denervation did not result in a functional change in the effectiveness of CL 316243 to reduce energy intake relative to sham- operated animals.

### 3.2. Study 2: Determine the Extent to Which OT-Induced Activation of Sympathetic Outflow to IBAT Contributes to Its Ability to Increase T_IBAT_ in Female HFD-Fed Rats

Following confirmation that there was no functional defect in the effectiveness of IBAT to respond to CL 316243-elicited stimulation of β3-AR (Study 1), the objective here was to examine whether OT-induced stimulation of T_IBAT_ requires intact SNS innervation of IBAT. Three of the sixteen rats available at study onset were euthanized during the course of the study and were excluded from the data analysis.

**Figure 2:**
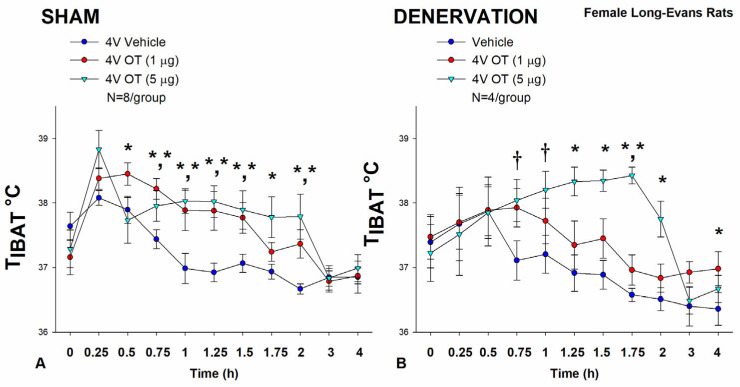
(**A**,**B**) **Effect of acute 4V OT administration (1 and 5 μg) on T_IBAT_ post-sham or IBAT denervation in female HFD-fed rats.** Rats were maintained on HFD (60% kcal from fat; N = 4–8/group) for approximately 4.5 months prior to undergoing a sham or bilateral surgical IBAT denervation and implantation of temperature transponders underneath IBAT. Rats were subsequently implanted with 4V cannulas and allowed to recover for 2 weeks prior to receiving acute 4V injections of OT or vehicle. Animals were subsequently adapted to a 4-h fast prior to receiving acute 4V injections of OT or vehicle (**A**,**B**) Effect of acute 4V OT on T_IBAT_ in (**A**) sham operated or (**B**) IBAT denervated DIO rats. Data are expressed as mean ± SEM. * *p* < 0.05, † 0.05 < *p* < 0.1 OT vs. vehicle.

In sham-operated rats, OT (5 μg) stimulated T_IBAT_ throughout the post-injection measurement period (0.75, 1, 1.25, 1.5, 1.75, and 2-h post-injection) (*p* < 0.05). In addition, the lower dose (1 μg) also stimulated T_IBAT_ throughout the post-injection measurement period (0.5, 0.75, 1, 1.25, and 1.5-h post-injection) (*p* < 0.05; Figure 2A).

Likewise, in IBAT denervated animals, OT (5 μg) stimulated T_IBAT_ throughout the post-injection measurement period (1.25, 1.5, 1.75, and 2-h post-injection) (*p* < 0.05) and also tended to increase T_IBAT_ at 0.75, and 1-h post-injection (0.05 < *p* < 0.1). The low dose (1 μg) increased T_IBAT_ at both 1.75 and 4-h post-injection (0.05 < *p* < 0.1; Figure 2B).

Notably, there was no significant change in the ability of 4V OT (5 μg) to increase T_IBAT_ when averaged over the 4-h post-injection period between sham and IBAT denervated animals (*p* = NS).

There were, however, seizures, barrel-rolling and unexpected deaths that occurred in three out of the sixteen rats (1 sham, 2 denervated) shortly after 4V administration of OT at the high dose (5 μg). Rinaman also reported that acute ICV administration of a higher dose (10 μg) also resulted in seizure-like activity and barrel-rolling in a subset of adult male Sprague-Dawley rats [68]. These findings raise the possibility that females may be more sensitive to the effects of acute injections of 4V OT compared to what we have observed previously at similar doses in males in the absence of such effects [34,46]. Thus, following the completion of these studies, we also examined the effectiveness of a lower dose of 4V OT (0.5 μg/μL) on T_IBAT_ in an identical manner.

**Figure 3:**
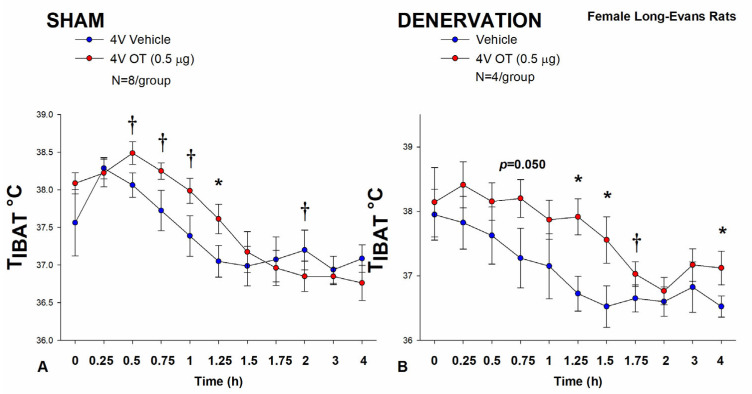
(**A**,**B**) **Effect of acute 4V OT administration (0.5 μg) on T_IBAT_ post-sham or IBAT denervation in female HFD-fed rats.** Rats were maintained on HFD (60% kcal from fat; N = 4–8/group) for approximately 4.5 months prior to undergoing a sham or bilateral surgical IBAT denervation and implantation of temperature transponders underneath IBAT. Rats were subsequently implanted with 4V cannulas and allowed to recover for 2 weeks prior to receiving acute 4V injections of OT or vehicle. Animals were subsequently adapted to a 4-h fast prior to receiving acute 4V injections of OT or vehicle (**A**,**B**) Effect of acute 4V OT on T_IBAT_ in (**A**) sham operated or (**B**) IBAT denervated DIO rats. Data are expressed as mean ± SEM. * *p* < 0.05, † 0.05 < *p* < 0.1 OT vs. vehicle.

In sham-operated animals, 4V administration of OT (0.5 μg/μL) increased T_IBAT_ at 1.25-h post-injection (*p* < 0.05; Figure 3A) and it also appeared to increase T_IBAT_ during the post-injection period (0.5, 0.75, and 1-h post-injection) (0.05 < *p* < 0.1; Figure 3A). Acute 4V administration of OT also tended to decrease T_IBAT_ at 2-h post-injection (0.05 < *p* < 0.1; Figure 3A).

In IBAT denervated animals, 4V OT (0.5 μg/μL) administration increased T_IBAT_ throughout the post-injection period (1.25, 1.5, and 4-h post-injection) (*p* < 0.05; Figure 3B). Acute 4V OT administration also tended to increase T_IBAT_ at both 0.75 (*p* = 0.050) and 1.75-h (*p* = 0.053) post-injection (Figure 3B). Acute 4V OT also stimulated T_IBAT_ at 24-h post-injection (*p* < 0.05).

Notably, there was no significant change in the ability of 4V OT (0.5 μg) to stimulate T_IBAT_ when averaged over the 1-h post-injection or at 1.25-h post-injection between sham and denervated rats (*p* = NS).

Collectively, these results indicate that IBAT denervation did not result in a functional change in the effectiveness of 4V OT administration to stimulate BAT thermogenesis in between denervated rats and sham-operated animals.

#### Plasma Hormone Concentrations

Here, we determined the effects of acute 4V OT (5 μg/μL) on plasma hormones in sham HFD-fed rats (Table 1). Samples from the denervated HFD-fed rats were excluded due to the lack of samples/group for valid comparisons (N = 1–2/group). There were no significant differences in any of the plasma measurements between vehicle and 4V OT-treated rats in the sham-operated groups.

### 3.3. Study 3A: Determine the Extent to Which OT-Induced Activation of Sympathetic Outflow to IBAT Contributes to Its Ability to Impact Body Weight in Female HFD-Fed Rats

The objective of Study 3A was to determine whether OT-evoked weight loss requires intact SNS outflow to IBAT. Initially, female rats were lean as defined by body weight (230 ± 2.1 g). As was the case with Study 1, HFD-fed rats were borderline obese as determined by both body weight (380 ± 8.3 g) and adiposity (126.4 ± 6.4 g fat mass; 32.8 ± 1.0% adiposity) after having been maintened on the HFD for at least 4.5 months prior to sham/denervation procedures.

Note that a subset of rats from Study 3 have been analyzed (9 out of 15) for IBAT NE content and all had successful IBAT denervation procedures. All 15 animals were included in the subsequent analyses. IBAT NE content was decreased by 83.1 ± 3.0% in a subset of denervated (9 out of 15) rats relative to a subset of sham-operated control rats (11 out of 15) [(F(1,18) = 64.663, *p* = 0.000)]. On the other hand, NE content was unchanged in other tissues, including IWAT, EWAT, liver or pancreas in denervated rats relative to sham rats (*p* = NS). As expected [35], there was no significant change in body weight between female sham-operated and IBAT denervated rats at the start of the study prior to minipump implantation (*p* = NS).

**Figure 4:**
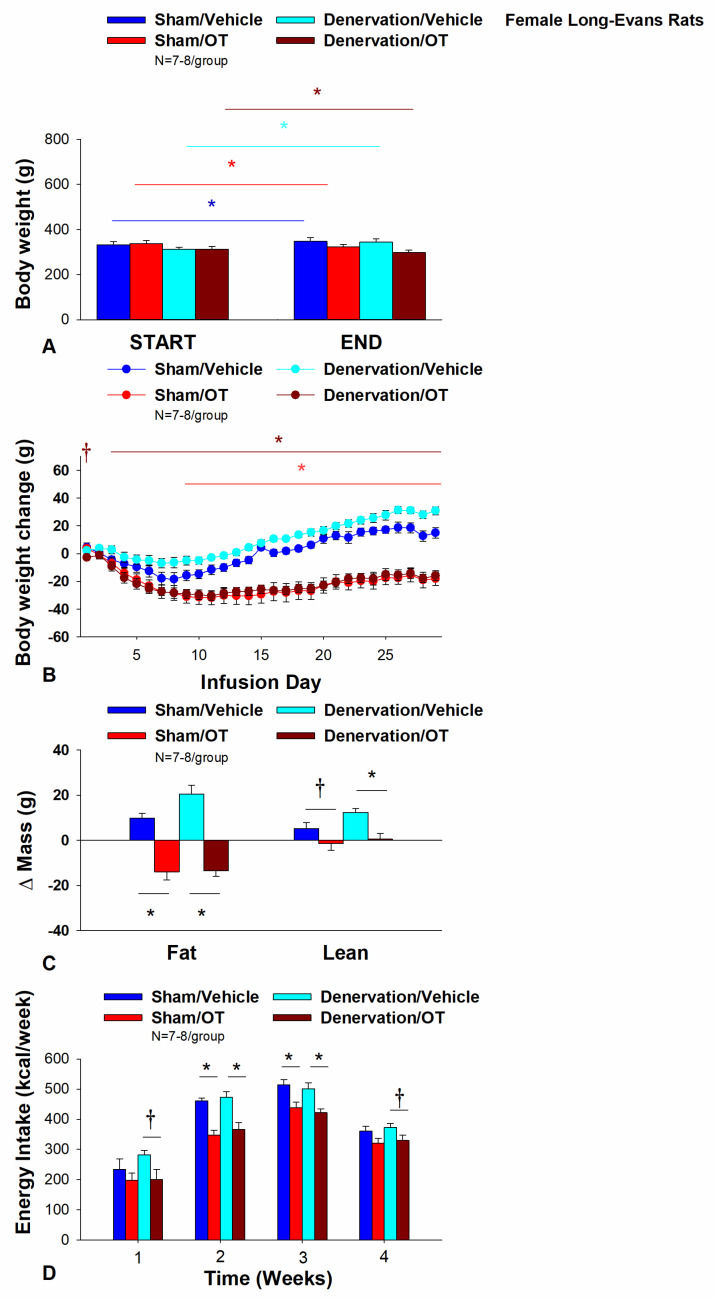
(**A**–**D**) **Effect of chronic 4V OT infusions (16 nmol/day) on body weight, adiposity and energy intake post-sham or IBAT denervation in female HFD-fed rats**. (**A**) Rats were maintained on HFD (60% kcal from fat; N = 7–8/group) for approximately 4.75–5.25 months prior to undergoing a sham or bilateral surgical IBAT denervation. Rats were subsequently implanted with 4V cannulas and allowed to recover for 2 weeks prior to being implanted with subcutaneous minipumps that were subsequently attached to the 4V cannula. (**A**) Effect of chronic 4V OT or vehicle on body weight in sham operated or IBAT denervated DIO rats; (**B**) Effect of chronic 4V OT or vehicle on body weight change in sham operated or IBAT denervated DIO rats; (**C**) Effect of chronic 4V OT or vehicle on adiposity in sham operated or IBAT denervated DIO rats; (**D**) Effect of chronic 4V OT or vehicle on adiposity in sham operated or IBAT denervated DIO rats. Data are expressed as mean ± SEM. * *p* < 0.05, † 0.05 < *p* < 0.1 OT vs. vehicle.

Chronic 4V administration of vehicle resulted in a 4.5 ± 1.2% weight gain compared to vehicle pre-treatment [(F(1,6) = 14.125, *p* = 0.009)] in female sham-operated rats. In contrast, chronic 4V OT treatment resulted in a 5.5 ± 1.3% reduction of body weight compared to 4V OT pre-treatment [(F(1,7) = 8.169, *p* = 0.024)] (Figure 4A). Chronic 4V OT treatment also decreased weight gain throughout the 29-day infusion period, specifically over treatment days 9–29 (*p* < 0.05; Figure 4B). At the conclusion of the study (infusion day 29), OT had decreased body weight by −18 ± 5.0 g in comparison with vehicle-treated rats (15.0 ± 3.8 g; *p* < 0.05). Chronic 4V OT treatment also decreased relative fat mass (pre- vs. post-intervention) (Figure 4C; *p* < 0.05), fat mass and relative lean mass (pre- vs. post-intervention) but had no impact on total lean body mass (*p* = NS). These effects that were associated with a modest decrease in energy intake that was apparent over treatment weeks 2 and 3 (Figure 4D; *p* < 0.05).

Chronic 4V vehicle treatment resulted in 8.9 ± 1.2% weight gain relative to vehicle pre-treatment in female IBAT denervated rats [(F(1,6) = 65.633, *p* = 0.000)]. In addition, 4V OT treatment also resulted in a 4.1 ± 1.0% reduction of body weight relative to 4V OT pre-treatment [(F(1,7) = 13.723, *p* = 0.008)]. (Figure 4A). Chronic 4V OT also reduced weight gain throughout the 29-day infusion period, specifically over treatment days 3–29 (*p* < 0.05; Figure 4B). At the conclusion of the study (infusion day 29), OT had decreased body weight by −15.9 ± 3.7 g in comparison with vehicle-treated rats (30 ± 2.9 g; *p* < 0.05). Chronic 4V OT treatment also decreased relative fat mass (pre- vs. post-intervention) (Figure 4C; *p* < 0.05) and fat mass (*p* < 0.05) but had no impact on total lean body mass (*p* = NS). These effects that were associated with a modest decrease in energy intake that was apparent during treatment weeks 2 and 3 (Figure 4D; *p* < 0.05). Chronic 4V OT treatment also tended to decrease energy intake during week 1 (*p* = 0.052) and 4 (*p* = 0.075) of the infusion period. There was also no significant effect of chronic 4V OT to increase kaolin intake over the course of the treatment period (*p* = NS).

Notably, there was no change in the ability of chronic 4V OT treatment to decrease body weight, energy intake, and relative fat mass (pre- vs. post-intervention) or fat mass between female sham-operated and IBAT denervated rats (*p* = NS).

#### T_IBAT_

Similar to what we have previously reported following chronic third ventricular (3V) [34] and 4V [52] treatment in male rats, chronic 4V administration of OT appeared to increase T_IBAT_ (at onset of light cycle) relative to vehicle treatment in female sham-operated rats. This was evident when the data were averaged over week 2 of the infusion period (Table 2A; *p* = 0.081) and throughout the infusion period in ad libitum fed rats on days 6 (*p* = 0.068), 12 (*p* < 0.05) and 21 (*p* < 0.05).

In order to minimize the confound of diet-induced thermogenesis, we collected T_IBAT_ from the same sham operated rats following a 4-h fast. Chronic 4V OT elevated T_IBAT_ when the data were averaged over week 1 of the infusion period (Table 2B; *p* = 0.066) and on infusion days 3 (*p* < 0.05), 5 (*p* < 0.05), and 7 (*p* < 0.05).

In addition, chronic 4V administration of OT also appeared to stimulate T_IBAT_ in denervated rats when the data were averaged over weeks 3 (Table 2C; *p* = 0.054) and 4 (Table 2C; *p* < 0.05) and throughout the infusion period on days 15 (*p* < 0.05), 18 (*p* = 0.063), 20 (*p* = 0.057), 21 (*p* = 0.050), 23 (*p* < 0.05), 24 (*p* < 0.05) and 26 (*p* < 0.05). In contrast, chronic 4V OT failed to elicit a change in T_IBAT_ in IBAT denervated rats that underwent a 4-h fast (Table 2D; *p* = NS).

Based on these findings as a whole, we conclude that SNS innervation of IBAT does not appear to be a predominant contributor of OT-elicited reduction of weight gain and adiposity in female HFD-fed rats.

### 3.4. Study 3B: Determine the Extent to Which 4V OT Impacts Thermogenic Gene Expression in IBAT and IWAT in Female HFD-Fed Rats

The objective of this study was to determine whether chronic 4V OT treatment stimulates thermogenic gene expression in IBAT and EWAT from female sham-operated rats.

#### 3.4.1. IBAT

We found that chronic 4V OT treatment elicited a near significant increase in β3-AR mRNA expression (*Adrb3*; *p* = 0.063) and a near significant reduction of Dio2 mRNA expression (*p* = 0.077; Table 3A).

#### 3.4.2. IWAT

4V OT treatment was associated with a significant increase of the thermogenic markers, beta 1 adrenergic receptor (β1-AR) (*Adrb1*; *p* < 0.05; Table 3B) and Cidea (*p* < 0.05) mRNA expression. 4V OT treatment also elicited a near significant increase in Gpr120 mRNA expression (*p* = 0.060) as well as a near significant reduction of Dio2 mRNA expression (*p* = 0.097) in IWAT from sham-operated rats.

Together, these findings raise the possibility that different thermogenic markers in IBAT and IWAT may contribute, in part, to the ability of chronic 4V OT to reduce body weight and adiposity in sham-operated rats.

### 3.5. Study 4A: Determine the Effects of Chronic 4V OT Treatment (16 nmol/day) on Body Weight, Adiposity and Energy Intake in Female DIO C57BL/6J Mice

The objective of this study was to determine the susceptibility of female C57BL/6J mice to DIO and whether the effects of chronic hindbrain (4V) administration to reduce body weight and adiposity could translate to another female rodent model (female C57BL/6J mice). Initially, female C57BL/6J mice were lean as determined by both body weight (17.4 ± 0.3 g) and adiposity (2.2 ± 0.2 g fat mass; 12.7 ± 0.8% adiposity). HFD-fed C57BL/6J mice became borderline DIO as demonstrated by both body weight (31.2 ± 1.4 g) and adiposity (11.7 ± 1.2 g fat mass; 36 ± 2.2% adiposity) after maintenance on the HFD for at least 4.5 months prior to being IBAT temperature transponder and minipump implantations as described earlier. By design, there was no significant difference in initial body weight or adiposity between vehicle and OT treatment groups prior to minipump implantation (*p* = NS). Three of the twenty mice available at study onset were euthanized during the course of the study and were excluded from the data analysis (including one whose head cap had become detached).

**Figure 5:**
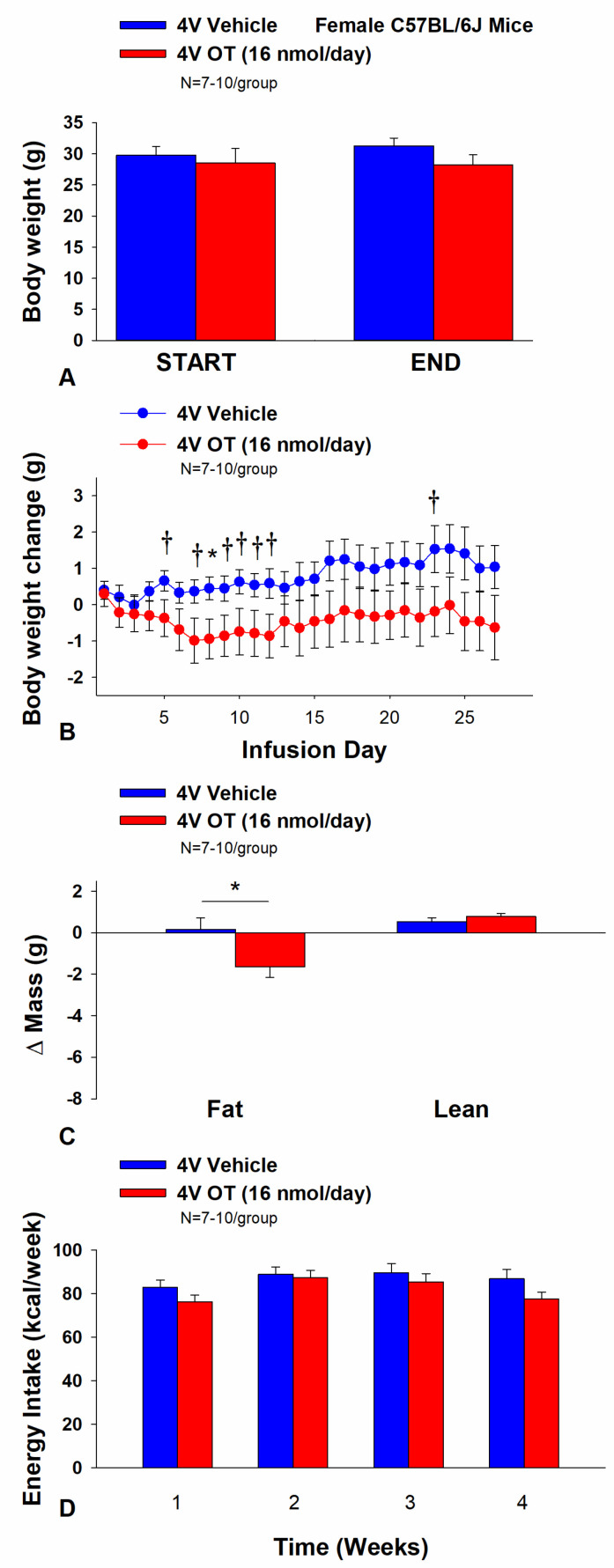
(**A**–**D**) **Effect of chronic 4V OT infusions (16 nmol/day) on body weight, adiposity and energy intake in female HFD-fed C57BL/6J mice.** (**A**) Mice were maintained on HFD (60% kcal from fat; N = 7–10/group) for approximately 4.5 months prior to implantation of temperature transponders underneath IBAT. Mice were subsequently implanted with 4V cannulas and allowed to recover for 2 weeks prior to being implanted with subcutaneous minipumps that were subsequently attached to the 4V cannula. (**A**) Effect of chronic 4V OT or vehicle on body weight in female C57BL/6J mice rats; (**B**) Effect of chronic 4V OT or vehicle on body weight change in female C57BL/6J mice; (**C**) Effect of chronic 4V OT or vehicle on adiposity in female C57BL/6J mice; (**D**) Effect of chronic 4V OT or vehicle on adiposity in female C57BL/6J mice. Data are expressed as mean ± SEM. * *p* < 0.05, † 0.05 < *p* < 0.1 OT vs. vehicle.

Chronic 4V vehicle treatment in female C57BL/6J mice resulted in modest amount of weight relative to vehicle pre-treatment (*p* = 0.114). While chronic 4V OT treatment failed to evoke weight loss (*p* = NS; Figure 5A), it reduced weight gain on treatment day 8 (*p* < 0.05) and it also tended to reduce weight gain on treatment days 5, 7, 9–12, and 23 (0.05 < *p* < 0.1; Figure 5B). These effects were associated with a reduction of relative fat mass (pre- vs. post-intervention) (Figure 5C; *p* < 0.05). Chronic 4V OT treatment also tended to reduce total lean mass (*p* = 0.131) but had no effect on relative lean mass (pre- vs. post-intervention) or total lean mass (*p* = NS). These effects were not associated with significant reductions in energy intake (Figure 5D; *p* = NS) or increased kaolin consumption (*p* = NS) during the course of the treatment period.

#### 3.5.1. T_IBAT_

In contrast to the effects of observed following chronic 4V infusions of OT (16 nmol/day) in male [54] and female DIO rats, we found that chronic 4V infusions of OT at the same dose (16 nmol/day) in female C57BL/6J mice largely had no effect significant effects on T_IBAT_ in ad libitum fed mice when the data were averaged over weeks 1, 2, 3 and 4 of the infusion period (*p* = NS).

While largely similar results were obtained following a 4-h fast over weeks 1–3 (*p* = NS), there was a tendency for chronic 4V OT to reduce T_IBAT_ over week 4 (*p* = 0.076) in 4-h fasted mice. Specifically, chronic 4V OT reduced T_IBAT_ on infusion days 17, 20, and 25 (*p* < 0.05) and tended to reduce T_IBAT_ on infusion days 13, 23, and 24 (0.05 < *p* < 0.1).

#### 3.5.2. Plasma Hormone Concentrations

Here, we determined the effects of chronic 4V OT (16 nmol/day) on plasma hormones in female C57BL/6J mice (Table 4). We did not find any significant differences in any of the plasma measurements in female C57BL/6J mice that received chronic 4V infusions of vehicle or OT.

### 3.6. Study 4B: Determine the Effects of Chronic 4V OT Treatment (16 nmol/day) on Body Weight, Adiposity and Energy Intake in Female DIO DBA/2J Mice

The objective of this study was to determine the susceptibility of female DMA/2J mice to DIO and whether the effects of chronic hindbrain (4V) administration to reduce body weight and adiposity could translate to another rodent model (female DBA/2J mice). This particular strain of female mice was previously found to be susceptible to becoming DIO [69,70]. Initially, female DBA/2J mice were lean as determined by both body weight (20.3 ± 0.3 g) and adiposity (3.4 ± 0.3 g fat mass; 16.8 ± 1.1% adiposity). DBA/2J mice became DIO as determined by both body weight (35.8 ± 0.9 g) and adiposity (15.1 ± 0.8 g fat mass; 41.9 ± 1.2% adiposity) after maintenance on the HFD for at least 4.5 months prior to IBAT temperature transponder and minipump implantations as described earlier. There was no significant difference in initial body weight or adiposity between vehicle and OT treatment groups prior to minipump implantation (*p* = NS). Four of the twenty mice available at study onset were euthanized during the course of the study and were excluded from the data analysis (including two whose head caps had become detached).

**Figure 6:**
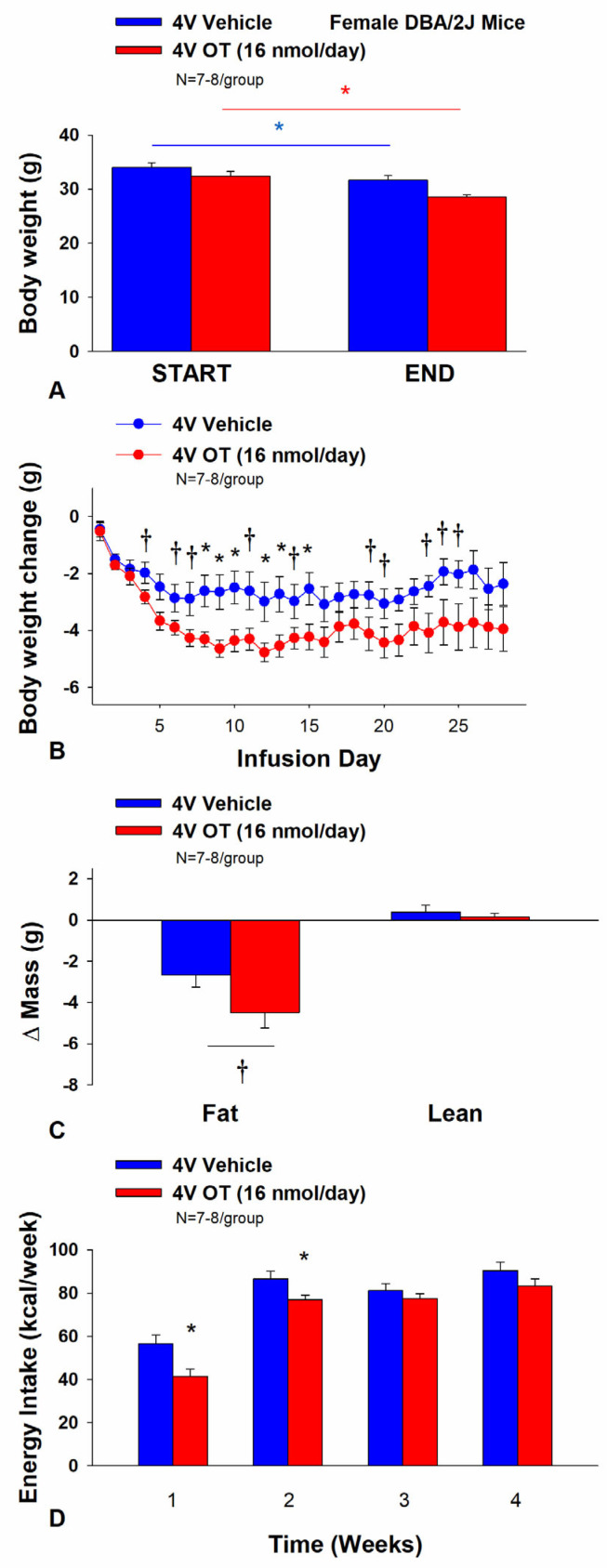
(**A**–**D**) **Effect of chronic 4V OT infusions (16 nmol/day) on body weight, adiposity and energy intake in female HFD-fed DBA/2J mice**. (**A**) Mice were maintained on HFD (60% kcal from fat; N = 7–8/group) for approximately 4.5 months prior to implantation of temperature transponders underneath IBAT. Mice were subsequently implanted with 4V cannulas and allowed to recover for 2 weeks prior to being implanted with subcutaneous minipumps that were subsequently attached to the 4V cannula. (**A**) Effect of chronic 4V OT or vehicle on body weight in female DBA/2J mice; (**B**) Effect of chronic 4V OT or vehicle on body weight change in female DBA/2J mice; (**C**) Effect of chronic 4V OT or vehicle on adiposity in female DBA/2J mice; (**D**) Effect of chronic 4V OT or vehicle on adiposity in female DBA/2J mice. Data are expressed as mean ± SEM. * *p* < 0.05, † 0.05 < *p* < 0.1 OT vs. vehicle.

Unexpectedly, chronic 4V vehicle resulted in 6.4 ± 2.0% weight loss relative to chronic 4V vehicle pre-treatment [(F(1,7) = 12.781, *p* = 0.009)] whereas chronic 4V OT reduced body weight by 11.9 ± 2.1% relative to chronic 4V OT pre-treatment [(F(1,6) = 24.802, *p* = 0.003)] (Figure 6A). Furthermore, chronic 4V OT treatment reduced weight gain on treatment days 8–10, 12–13, and 15 (*p* < 0.05) and it also tended to reduce weight gain on treatment days 4, 5 (*p* = 0.050), 6–7, 11, 14, 19–21, and 23–25 (0.05 < *p* < 0.1; Figure 6B). Chronic 4V OT also tended to reduce relative fat mass (pre- vs. post-intervention) (Figure 6C; 0.05 < *p* < 0.1) and total fat mass (*p* < 0.05) but had no effect on adipocyte size or relative lean mass (pre- vs. post-intervention) or total lean mass (*p* = NS). These effects were associated with a modest reduction of energy intake that was apparent during weeks 1 and 2 of the treatment period (Figure 6D; *p* < 0.05). There was no effect of chronic 4V OT to increase kaolin consumption during the treatment period (*p* = NS).

#### 3.6.1. T_IBAT_

Chronic 4V infusions of OT (16 nmol/day) had no significant effect on T_IBAT_ in ad libitum fed mice when the data were averaged over weeks 1, 2, 3 and 4 (*p* = NS).

While similar results were obtained following a 4-h fast over weeks 1–4 (*p* = NS), chronic 4V OT reduced T_IBAT_ on infusion day 8 (*p* < 0.05) and tended to reduce T_IBAT_ on infusion day 26 (0.05 < *p* < 0.1).

#### 3.6.2. Plasma Hormone Concentrations

Here, we determined the effects of chronic SC OT (16 and 50 nmol/day) on plasma hormones in female DBA/2J mice (Table 5). We did not find any significant differences in any of the plasma measurements in female DBA/2J mice that received chronic 4V infusions of vehicle or OT.

### 3.7. Study 5: Determine the Effects of Chronic Systemic OT Treatment (16 and 50 nmol/day) on Body Weight, Adiposity and Energy Intake in Female DIO DBA/2J Mice

The objective of this study was to extend previous findings from Study 4B and determine whether SC infusion of a centrally effective dose of OT (16 nmol/day) can decrease both body weight and adiposity in female DIO DBA/2J mice (same strain used in Study 4B). DBA/2J mice became DIO as determined by the increased body weight (32.2 ± 0.9 g) and adiposity (12.7 ± 0.7 g fat mass; 38.7 ± 1.2% adiposity) after being maintained on the HFD for at least 4.5 months prior to IBAT temperature transponder implantations. By design, there was no significant difference in body weight or adiposity between vehicle and OT treatment groups at the start of the study prior to minipump implantation (*p* = NS).

**Figure 7:**
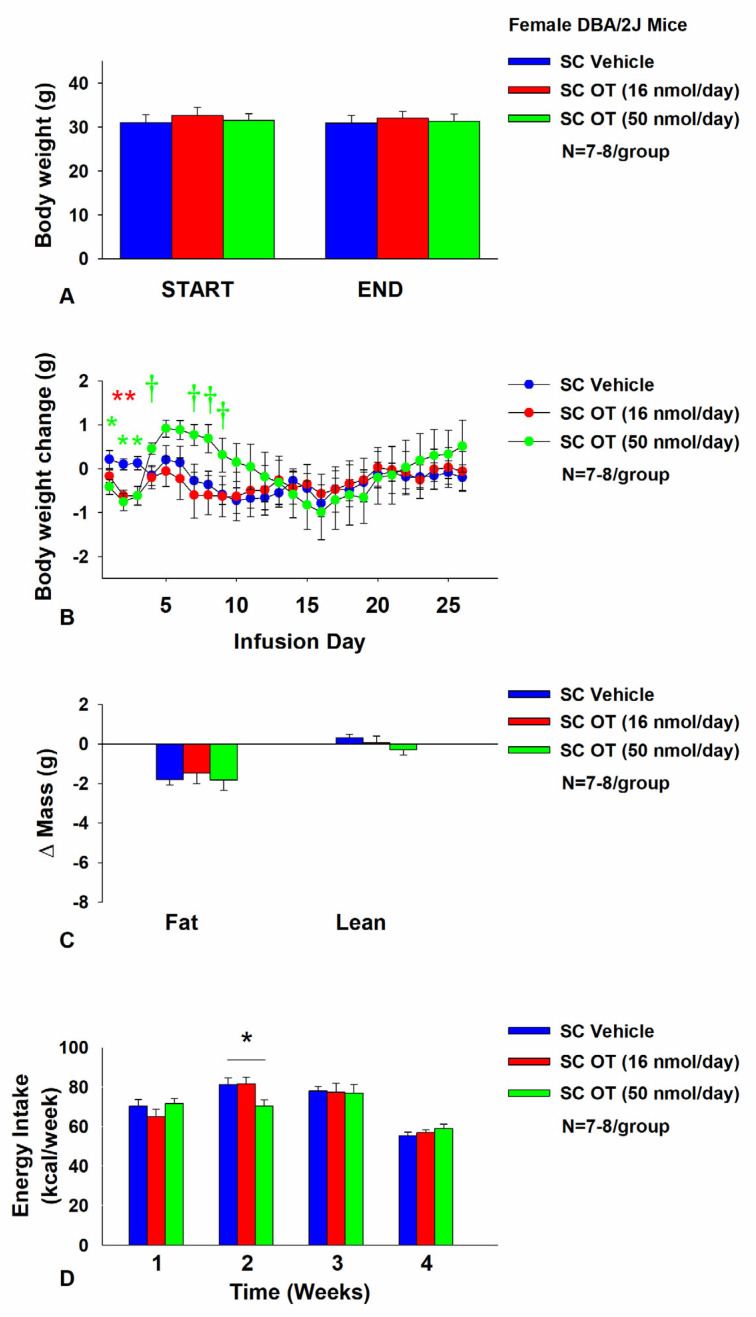
(**A**–**D**) Effect of chronic systemic OT infusions (16 and 50 nmol/day) on body weight, adiposity and energy intake in female HFD-fed DBA/2J mice. (**A**) Mice were maintained on HFD (60% kcal from fat; N = 7–8/group) for approximately 4.5 months prior to implantation of temperature transponders underneath IBAT. Mice were subsequently implanted with 4V cannulas and allowed to recover for 2 weeks prior to being implanted with SC minipumps that were subsequently attached to the 4V cannula. (**A**) Effect of chronic 4V OT or vehicle on body weight in female DBA/2J mice; (**B**) Effect of chronic 4V OT or vehicle on body weight change in female DBA/2J mice; (**C**) Effect of chronic 4V OT or vehicle on adiposity in female DBA/2J mice; (**D**) Effect of chronic 4V OT or vehicle on adiposity in female DBA/2J mice. Data are expressed as mean ± SEM. * *p* < 0.05, † 0.05 < *p* < 0.1 OT vs. vehicle.

In contrast to chronic 4V OT treatment in female DBA/2J mice (Study 4B), chronic SC OT treatment did not result in a significant reduction of body weight (Figure 7A). SC OT treatment (16 nmol/day) reduced weight gain on treatment days 2–3 (*p* < 0.05) and tended to reduce weight gain on treatment day 1 (*p* = 0.138; Figure 7B). The higher dose (50 nmol/day) also reduced weight gain on treatment days 1–3 and tended to increase body weight gain on treatment days 4 (*p* = 0.055), 5 (*p* = 0.106), 7 (*p* = 0.077), 8 (*p* = 0.051) and 9 (*p* = 0.092) (Figure 7B). There was no effect of SC OT at either dose on relative fat mass or lean mass (pre- vs. post-intervention) (Figure 7C). OT (50 nmol/day) produced a transient reduction of energy intake during week 2 (Figure 7D; *p* < 0.05) but OT failed to impact energy intake at any other time. There was also no effect of chronic SC OT to significant increase kaolin consumption during weeks 2–4 of the treatment period (*p* = NS) but a slight reduction of kaolin intake during week 1 in response to the higher dose 50 nmol/day (*p* = 0.016).

#### 3.7.1. T_IBAT_

In contrast to what we found following chronic 4V administration, chronic SC administration of OT (16 and 50 nmol/day) reduced T_IBAT_ relative to vehicle in ad libitum fed mice when the T_IBAT_ data were averaged over weeks 3 and 4 (Table 6A; *p* < 0.05) of the infusion period. Similar results were obtained from the same mice following a 4-h fast over the same period (Table 6B; *p* < 0.05).

In addition to the findings in DIO female DBA/2J mice, we found that there appeared to be a very modest effect of chronic systemic OT (16 nmol/day) to reduce T_IBAT_ in 4-h fasted male DIO (C57Bl/6J) mice on infusion day 9 (*p* < 0.05) and tended to reduce T_IBAT_ on days 10 (0.05 < *p* < 0.1), 14 (0.05 < *p* < 0.1), and 21 (*p* = 0.05) (unpublished findings). Likewise, OT (50 nmol/day) tended to reduce T_IBAT_ in 4-h fasted C57Bl/6J mice on infusion day 4 (*p* < 0.05) and tended to reduce T_IBAT_ on infusion day 21 (0.05 < *p* < 0.1). In contrast, we found that OT (100 nmol/day) tended to increase T_IBAT_ on infusion day 13 (0.05 < *p* < 0.1), but this was only evident in ad libitum fed C57Bl/6J mice but not in 4-h fasted mice.

#### 3.7.2. Plasma Hormone Concentrations

Here, we examined the effects of chronic systemic OT (16 and 50 nmol/day) on plasma hormones in female DBA/2J mice (Table 7). Chronic SC OT (16 nmol/day) treatment was associated with a significant increase in plasma glucagon in female DBA/2J mice. Chronic SC OT treatment also tended to increase total cholesterol at the low (16 nmol/day; *p* = 0.057) and high dose (50 nmol/day; *p* = 0.062). In addition, chronic SC Ot at the high dose (50 nmol/day) also tended to produce an increase in plasma leptin (*p* = 0.092). and FGF-21 (*p* = 0.051).

## 4. Discussion

The objectives of the current set of studies were to (1) establish whether sympathetic innervation of IBAT is required for 4V (hindbrain) administration of OT to stimulate BAT thermogenesis and decrease body weight and adiposity in female HFD-fed rats and (2) establish whether the ability of hindbrain (4V) infusion of OT to elicit weight loss translates to other rodent species. To accomplish these goals, we examined the effect of disrupting SNS activation of IBAT on OT-induced stimulation of T_IBAT_ and reduction of body weight in HFD-fed rats. We initially determined the impact of bilateral surgical SNS denervation to IBAT on the ability of acute 4V OT (0.5, 1, and 5 µg) to stimulate T_IBAT_ in female HFD-fed rats. We found that the high dose of 4V OT (5 µg) stimulated T_IBAT_ similarly between sham rats and denervated rats. We subsequently determined if OT-elicited reductions of body weight and adiposity require intact SNS outflow to IBAT. To accomplish this, we determined the effect of bilateral surgical or sham denervation of IBAT on the ability of chronic 4V OT (16 nmol/day) or vehicle administration to reduce body weight, adiposity and food intake in female HFD-fed rats. Chronic 4V OT reduced body weight gain (sham: −18.0 ± 4.9 g; denervation: −15.9 ± 3.7 g) and adiposity (sham: −13.9 ± 3.7 g; denervation: −13.6 ± 2.4 g) relative to vehicle treatment and these effects were similar between groups. These effects were attributed, in part, to reduced energy intake evident during weeks 2 and 3. To test whether the effects of 4V OT to elicit weight loss translate to other female rodent species, we also examined the effect of chronic 4V infusion of OT on body weight in two separate strains of female HFD-fed mice. Similar to what we found in the HFD-fed rat model, we also found that chronic 4V OT (16 nmol/day) infusion resulted in reduced body weight gain, adiposity and/or energy intake in female HFD-fed C57BL/6J and DBA/2J mice. Together, these findings suggest that (1) sympathetic innervation of IBAT is not necessary for OT-elicited increases in BAT thermogenesis and weight loss in female HFD-fed rats and (2) the effects of OT to reduce weight gain and adiposity translate to other female mouse models of diet-induced obesity (DIO).

We have now determined that chronic 4V OT-elicited reduction of body weight loss does not require SNS innervation of IBAT in multiple animal models (female HFD-fed rats and male DIO mice) [35]. These data suggest that 4V administration of OT increases BAT thermogenesis and evokes weight loss through a mechanism that does not require SNS innervation of IBAT in male and female rodent models. As mentioned in [35], we have not addressed what mechanism might be required for hindbrain (4V) OT to stimulate BAT thermogenesis if not through SNS innervation of IBAT. We have largely ruled out the possibility that, in mice, 4V OT might be leaking into the periphery to act at peripheral OTRs by showing that systemic administration of OT, at a centrally effective dose was unable to replicate the effects of hindbrain (4V) OT to reduce body weight and stimulate BAT thermogenesis in DIO mice [35]. One mechanism that we did not address in this body of work is whether 4V OT-elicited activation of hindbrain and/or spinal cord OTRs might elicit the release of epinephrine from the adrenal medulla and activate BAT thermogenesis through direct activation of β-adrenergic receptors. However, we recently determined that systemic administration of the β3-AR antagonist, SR 59230A, failed to block the effects of acute 4V OT to increase T_IBAT_ (unpublished findings) in male DIO Long-Evans rats, suggesting that signaling through the β3-AR is not required for OT-elicited BAT thermogenesis. Other potential mediators of 4V-OT elicited BAT thermogenesis include other beta-receptor subtypes, namely the β1-AR and β2-AR, both of which are expressed in IBAT in both mice [71] and rats [72,73]. While we did not find a significant increase in β1-AR mRNA expression in response to 4V OT in IBAT in this study, we did see an increase in β1-AR mRNA expression in IWAT (see discussion below). It is well appreciated that the β1-AR is important in the control of thermogenesis in rodents [74,75] but the β2-AR may be more important in the control of thermogenesis in humans than rodents [76,77,78]. Furthermore, β1-AR and β2-AR have nearly equal affinity for L-epinephrine in Chinese hamster ovary cells [79] and epinephrine administration to brown adipocytes stimulates fatty acids and respiration [80]. However, only 1% of parvocellular or magnocellular PVN OT neurons have poly-synaptic projections to the adrenal gland [81], despite the hindbrain and spinal cord being relay sites in outgoing poly-synaptic projections to the adrenal gland [81,82]. Future studies should address whether adrenal demedulation impairs the ability of hindbrain (4V) OT to stimulate BAT thermogenesis and elicit weight loss in DIO rodents.

Our finding that 4V OT treatment elicited an increase in both β1-AR and Cidea mRNA expression in IWAT raises the possibility that WAT browning or beiging may also contribute, in part, to the metabolic effects of 4V OT in female rodents. Beige depots within WAT may account for up to 5% of total UCP-1 [83,84]. It is possible that hindbrain OTRs could also be a component of descending projections that originate in the PVN and are important in the regulation of SNS outflow to IWAT [85]. In fact, there are well established poly-synaptic circuits that link parvocellular PVN OT neurons to IWAT [86,87]. Thus, OT neurons within the parvocellular PVN are anatomically situated to control WAT thermogenesis. One outstanding question is whether these effects are mediated by parvocellular PVN OT neurons that project directly to the hindbrain (nucleus tractus solitarius [88,89]) and/or spinal cord [89]. Further studies that determine the extent to which 4V OT treatment (1) elicits more functional changes in IWAT thermogenesis (increased temperature of IWAT) [85,90] and (2) reduces body weight and adiposity in animals following IWAT denervation will be helpful in assessing the role of WAT in contributing to the effects of 4V OT to reduce body weight and adiposity.

We acknowledge the possibility that the effects of 4V OT on BAT thermogenesis in female rats could be due, in part, to increased activity-induced thermogenesis [21] as well as skeletal muscle thermogenesis [22,23]. While we did not assess the effects of 4V OT on non-shivering and shivering thermogenesis in skeletal muscle [22,23] in this study, we recently determined that acute 4V administration of OT (5 μg) stimulated T_IBAT,_ core temperature and gross motor activity in male DIO rats (unpublished observations). However, we found that 4V OT-associated elevations of T_IBAT_ and core temperature occurred before significant increases in gross motor activity suggesting that changes in gross motor activity are not likely tied to the changes in T_IBAT_ and core temperature that preceded changes in activity. Our findings are similar to what others have reported following ICV (0.5 μg) [91] and ventromedial hypothalamic administration (1 nmol ≈ 1.0072 μg) [15]. Taken together, acute CNS administration of OT can increase activity in rodents, but, based on our unpublished findings, these activity related increases do not appear to contribute to the effects of 4V OT on BAT thermogenesis in male DIO rats. It remains to be determined if this holds true in female HFD-fed rats.

One limitation to this study is that we did not account for the contribution of other BAT depots in contributing to the ability of 4V OT to stimulate BAT thermogenesis and reduce body weight in IBAT denervated rats. We chose to make IBAT the focus of our studies given that it contains up to 45% of total UCP-1 [92] and represents ≥70% of total BAT mass [93]. In addition, this particular depot is the best characterized of BAT depots [94]. However, other BAT depots [axillary (subscapular), cervical, mediastinal and perirenal depots] show cold-induced elevations of UCP-1 [83]. In particular, the axillary (subscapular), cervical, periaortic and perirenal BAT depots [19,84] may provide up to 50% of total UCP-1 mRNA. Fischer reported that the axillary (subscapular) depot, also showed a significant 2-fold increase of total UCP-1 (UCP-1/scBAT) in response to HFD (diet-induced thermogenesis) in IBAT denervated mice [92]. There also appeared to be an increase of axillary UCP-1 in response to HFD in sham mice but it was not significant and there were no significant differences in UCP-1 between sham vs. denervation groups in response to HFD [92]. Moreover, Nugyen reported that is potential crosstalk between SNS circuits that innervate IBAT and WAT [85]. Nguyen found that there is increased NE turnover and IWAT UCP-1 mRNA expression in hamsters following SNS denervation of IBAT [85]. It will be helpful to selectively denervate other BAT and WAT depots in order to determine whether these depots may contribute, in part, to the effects of 4V OT to reduce body weight gain in female rodents.

While a recent study reported that systemic infusions of OT (100 nmol/day) result in an elevation of core temperature and increased IBAT gene expression in male HFD-fed mice (C57BL6/J) [38], we found that systemic infusion (16 and 50 nmol/day) resulted in a reduction of T_IBAT_ temperature in female DBA/2J mice. Similarly, we found that acute peripheral administration of OT (5 and 10 μg/μL) elicited an initial reduction of T_IBAT_ prior to a subsequent elevation of T_IBAT_ [35]. Furthermore, others have found that systemic injections of higher doses (1 mg/kg) have also resulted in hypothermic effects [95], which is thought to be mediated, in part, by activation of arginine vasopressin receptor 1A (AVPR1A) [96]. It is possible that differences between our study and Yuan’s study are due, in part, to strain, sex, age, length of time that the mice were maintained on the HFD prior to study onset (8 weeks rather than 18 weeks in our study) and/or time of day that the core temperature vs. T_IBAT_ measurements were taken. Being able to include measurements of core temperature and T_IBAT_ from the same animal will help enable more direct comparisons with other studies.

Based on recent findings [10], it is possible that differences in estrus cycle might have impaired the effectiveness of OT to reduce food intake during the measurement period. The authors found that there was an impaired ability of ICV OT to reduce food intake during the pro-estrus stage of the estrus cycle, during which time there is an increase in estrogen [10]. Despite this, we still found an effect of 4V OT to reduce weight gain suggesting that other mechanisms (i.e., lipolysis, energy expenditure) may also contribute to 4V OT-elicited changes in body weight in female rodents. Future studies, however, should take into account estrus cycle when measuring energy intake in response to OT treatment.

Our findings showing that chronic 4V administration of OT reduced energy intake in female DIO DBA/2J mice recapitulated the effects an earlier study that found following chronic systemic administration in female DIO C57BL/6J mice [97]. However, we failed to find an effect of chronic 4V OT to reduce food intake in female DIO C57BL/6J mice. In addition, we found that systemic OT (16 or 50 nmol/day) produced transient reductions of body weight gain in female DIO DBA/2J mice at doses that the authors (≈27.6 and 55.1 nmol/day) found to reduce body weight in female DIO C57BL/6J mice [97]. However, the authors in that study used a different strain of mice (C57BL/6J) that were younger (18 weeks vs. 31 weeks at onset of minipump infusions in our study), heavier (34.20 g vs. 31.2 ± 1.4 g in our study) and had been on the HFD diet for a shorter period of time (12 weeks vs. 24 weeks at onset of minipump infusions in our study). Thus, there are several differences between studies that might account for the contradictory effects.

In conclusion, our findings indicate that there is no significant difference in the effectiveness of the β3-AR agonist, CL 316243, to stimulate IBAT in female IBAT denervated rats relative to female sham-operated rats with intact SNS innervation of IBAT. In addition, we found that acute 4V administration of OT at both the low (0.5 µg) and high dose (5 µg) resulted in similar increases in T_IBAT_ at in female sham and IBAT denervated rats. Furthermore, we also found that there was no difference in the effectiveness of chronic 4V OT (16 nmol/day) to reduce body weight gain and adiposity in female sham and IBAT denervated rats. Consistent with what we found in the HFD-fed rat model, we found that chronic 4V OT (16 nmol/day) treatment reduced body weight gain, adiposity and/or energy intake in female DIO C57BL/6J and DBA/2J mice relative to chronic 4V vehicle treatment in control mice. Together, these findings suggest that (1) sympathetic innervation of IBAT is not required for OT to increase BAT thermogenesis and reduce body weight in female HFD-fed rats and (2) chronic hindbrain (4V) administration of OT reduces weight gain and adiposity in two different strains of female HFD-fed mice.

## Figures and Tables

**Table 1:** Plasma measurements following acute injections of 4V OT (5 μg/μL) or vehicle in female sham and IBAT denervated DIO rats. Data are expressed as mean ± SEM. (N = 3–4/group).

4V Treatment	Vehicle	OT
	Sham	Sham
Leptin (ng/mL)	22.2 ± 6.0 ^a^	20.7 ± 5.7 ^a^
Insulin (ng/mL)	2.6 ± 1.3 ^a^	2.4 ± 0.7 ^a^
Glucagon (pmol/L)	6.0 ± 0.3 ^a^	18.1 ± 8.9 ^a^
FGF-21 (pg/mL)	218.3 ± 71.3 ^a^	392.6 ± 172.6 ^a^
Irisin (mg/mL)	3.1 ± 0.7 ^a^	4.6 ± 0.9 ^a^
Adiponectin (mg/mL)	6.9 ± 0.7 ^a^	8.3 ± 0.8 ^a^
Blood Glucose (mg/dL)	149.3 ± 3.5 ^a^	138 ± 6.8 ^a^
FFA (mEq/L)	0.4 ± 0.1 ^a^	0.6 ± 0.1 ^a^
Total Cholesterol (mg/dL)	88.3 ± 12.4 ^a^	85.0 ± 8.8 ^a^

Blood was collected by tail vein nick (blood glucose) or from the trunk following a 6-h fast. Different letters denote significant differences between treatments. Shared letters are not significantly different from one another.

**Table 2:** Changes in T_IBAT_ following 4V infusions of OT or vehicle in female sham or IBAT denervated DIO rats. (**A**), Changes in T_IBAT_ following 4V infusions of OT or vehicle in ad libitum fed female sham or IBAT denervated DIO rats; (**B**), Changes in T_IBAT_ following 4V infusions of OT or vehicle in 4-h fasted female sham or IBAT denervated DIO rats. (**C**) Changes in TIBAT following 4V infusions of OT or vehicle in ad libitum fed female HFD-fed IBAT denervated rats. (**D**) Changes in TIBAT following 4V infusions of OT or vehicle in 4-h fasted female HFD-fed IBAT denervated rats. Data are expressed as mean ± SEM. * *p* < 0.05 OT, † 0.05 < *p* < 0.1 OT vs. vehicle (N = 7–8/group).

**Table 2A Changes in T_IBAT_ following 4V infusions of OT or vehicle in ad libitum fed** **female HFD-fed sham rats**				
**4V**	**Week 1**	**Week 2**	**Week 3**	**Week4**
**SHAM**	**Temp (°C)**	**Temp (°C)**	**Temp (°C)**	**Temp (°C)**
**Vehicle**	37.5 ± 0.3	37.5 ± 0.3	37.7 ± 0.3	37.5 ± 0.3
**OT**	37.9 ± 0.2	38.0 ± 0.2 ^†^	38.0 ± 0.1	37.9 ± 0.2
**Table 2B Changes in T_IBAT_ following 4V infusions of OT or vehicle in 4-h fasted** **fasted female HFD-fed sham rats**				
**4V**	**Week 1**	**Week 2**	**Week 3**	**Week4**
**SHAM**	**Temp (°C)**	**Temp (°C)**	**Temp (°C)**	**Temp (°C)**
**Vehicle**	37.7 ± 0.3	37.8 ± 0.2	37.6 ± 0.2	37.7 ± 0.3
**OT**	38.2 ± 0.2 ^†^	37.56 ± 0.3	37.5 ± 0.3	37.7 ± 0.1
**Table 2C Changes in T_IBAT_ following 4V infusions of OT or vehicle in ad libitum fed** **female HFD-fed IBAT denervated rats**				
**4V**	**Week 1**	**Week 2**	**Week 3**	**Week4**
**DENERVATION**	**Temp (°C)**	**Temp (°C)**	**Temp (°C)**	**Temp (°C)**
**Vehicle**	37.4 ± 0.1	37.3 ± 0.2	37.3 ± 0.1	37.1 ± 0.3
**OT**	37.6 ± 0.2	37.5 ± 0.1	37.9 ± 0.2 ^†^	37.8 ± 0.2 *
**Table 2D Changes in T_IBAT_ following 4V infusions of OT or vehicle in 4-h fasted** **female HFD-fed IBAT denervated rats**				
**4V**	**Week 1**	**Week 2**	**Week 3**	**Week4**
**DENERVATION**	**Temp (°C)**	**Temp (°C)**	**Temp (°C)**	**Temp (°C)**
**Vehicle**	38.1 ± 0.1	37.4 ± 0.3	37.7 ± 0.2	37.9 ± 0.2
**OT**	38.0 ± 0.1	37.5 ± 0.2	37.6 ± 0.2	37.6 ± 0.3

**Table 3:** (**A**,**B**). Changes in IBAT and IWAT gene expression following 4V infusions of OT or vehicle in female sham or IBAT denervated DIO rats. (**A**), Changes in IBAT mRNA expression 4V infusions of OT or vehicle in female sham or IBAT denervated DIO rats; (**B**), Changes in IWAT mRNA expression 4V infusions of OT or vehicle in female sham or IBAT denervated DIO rats. Shared letters are not significantly different from one another. Data are expressed as mean ± SEM (N = 7–8/group).

**Table 3A. Changes in IBAT mRNA Expression Following Chronic 4V Infusions of OT or Vehicle in Female HFD-Fed Rats**		
**4V Treatment**	**Vehicle**	**OT**
	**Sham**	**Sham**
**IBAT**		
*Adrb1*	1.0 ± 0.4 ^a^	1.5 ± 0.4 ^a^
*Adrb3*	1.0 ± 0.3 ^a^	1.9 ± 0.3 ^a^
*Ucp1*	1.0 ± 0.3 ^a^	1.2 ± 0.2 ^a^
*Cidea*	1.0 ± 0.3 ^a^	1.3 ± 0.3 ^a^
*Dio2*	1.0 ± 0.4 ^a^	0.3 ± 0.2 ^a^
*Gpr120*	1.0 ± 0.5 ^a^	1.2 ± 0.4 ^a^
*Prdm16*	1.0 ± 0.3 ^a^	1.2 ± 0.2 ^a^
*Ppargc1a*	1.0 ± 0.3 ^a^	1.2 ± 0.2 ^a^
**Table 3B. Changes in IWAT mRNA Expression Following Chronic 4V Infusions of OT or Vehicle in Female HFD-Fed Rats**		
**4V Treatment**	**Vehicle**	**OT**
	**Sham**	**Sham**
**IWAT**		
*Adrb1*	1.0 ± 0.2 ^a^	2.7 ± 0.6 ^b^
*Adrb3*	1.0 ± 0.4 ^a^	2.0 ± 0.5 ^a^
*Ucp1*	1.0 ± 0.3 ^a^	0.5 ± 0.1 ^a^
*Cidea*	1.0 ± 0.2 ^a^	2.8 ± 0.5 ^b^
*DIio2*	1.0 ± 0.3 ^a^	1.0 ± 0.2 ^a^
*Gpr120*	1.0 ± 0.3 ^a^	2.0 ± 0.4 ^a^
*Prdm16*	1.0 ± 0.1 ^a^	1.6 ± 0.3 ^a^
*Ppargc1a*	1.0 ± 0.2 ^a^	0.9 ± 0.1 ^a^

IBAT was collected following a 4-h fast. Different letters denote significant differences between treatments. Shared letters are not significantly different from one another. N = 7–8/group. IWAT was collected following a 4-h fast. Different letters denote significant differences between treatments. Shared letters are not significantly different from one another.

**Table 4:** Plasma measurements following chronic 4V infusions of OT (16 nmol/day) or vehicle in female HFD-fed C57BL/6J mice. Data are expressed as mean ± SEM (N = 7–10/group).

4V Treatment	Vehicle	OT
**Leptin (ng/mL)**	10.0 ± 1.3 ^a^	6.7 ± 1.3 ^a^
**Insulin (ng/mL)**	0.5 ± 0.1 ^a^	0.3 ± 0.04 ^a^
**Glucagon (pmol/L)**	23.3 ± 3.7 ^a^	34.1 ± 13.2 ^a^
**FGF-21 (pg/mL)**	467.5 ± 129.7 ^a^	513.6 ± 179.6 ^a^
**Irisin (mg/mL)**	4.1 ± 0.2 ^a^	3.9 ± 0.2 ^a^
**Adiponectin (mg/mL)**	19.3 ± 1.0 ^a^	20.1 ± 1.8 ^a^
**Blood Glucose (mg/dL)**	155 ± 2.7 ^a^	152.6 ± 8.5 ^a^
**FFA (mEq/L)**	0.13 ± 0.01 ^a^	0.11 ± 0.01 ^a^
**Total Cholesterol (mg/dL)**	105.9 ± 2.9 ^a^	95.3 ± 9.5 ^a^

Blood was collected by tail vein nick (blood glucose) or from the trunk following a 6-h fast. Different letters denote significant differences between treatments. Shared letters are not significantly different from one another.

**Table 5:** Plasma Measurements Following 4V Infusions of OT or Vehicle in Female HFD-Fed DBA/2J Mice.

4V Treatment	Vehicle	OT
**Leptin (ng/mL)**	5.1 ± 1.4 ^a^	3.1 ± 0.5 ^a^
**Insulin (ng/mL)**	2.6 ± 0.4 ^a^	1.4 ± 0.2 ^b^
**Glucagon (pmol/L)**	46.1 ± 11.2 ^a^	46.9 ± 8.7 ^a^
**FGF-21 (pg/mL)**	1419.1 ± 251.3 ^a^	1297.8 ± 282 ^a^
**Irisin (mg/mL)**	5.0 ± 0.2 ^a^	4.2 ± 0.3 ^a^
**Adiponectin (mg/mL)**	8.1 ± 0.2 ^a^	8.4 ± 0.3 ^a^
**Blood Glucose (mg/dL)**	140 ± 4.3 ^a^	145.4 ± 4.2 ^a^
**FFA (mEq/L)**	0.2 ± 0.02 ^a^	0.3 ± 0.1 ^a^
**Total Cholesterol (mg/dL)**	96.4 ± 4.1 ^a^	103.4 ± 5.2 ^a^

Blood was collected by tail vein nick (blood glucose) or from the trunk following a 6-h fast. Different letters denote significant differences between treatments. Shared letters are not significantly different from one another. Data are expressed as mean ± SEM (N = 7–8/group).

**Table 6:** Changes in T_IBAT_ following chronic systemic infusions of OT (16 and 50 nmol/day) or vehicle in female DBA/2J mice. A, Changes in T_IBAT_ following chronic systemic infusions of OT (16 and 50 nmol/day) or vehicle in ad libitum fed female DBA/2J mice; B, Changes in T_IBAT_ following chronic systemic infusions of OT (16 and 50 nmol/day) or vehicle in 4-h fasted female DBA/2J mice. Shared letters are not significantly different from one another. Data are expressed as mean ± SEM. * *p* < 0.05 OT, vs. vehicle (N = 7–8/group).

**Table 6A Changes in T_IBAT_ following SC infusions of OT or vehicle in ad libitum fed** **female DIO DBA/2J mice**				
**SC**	**Week 1**	**Week 2**	**Week 3**	**Week4**
	**Temp (°C)**	**Temp (°C)**	**Temp (°C)**	**Temp (°C)**
**Vehicle**	37.5 ± 0.2	37.1 ± 0.2	37.0 ± 0.1	37.2 ± 0.1
**OT (16 nmol/day)**	37.2 ± 0.2	36.7 ± 0.2	36.5 ± 0.2 *	36.4 ± 0.2 *
**OT (50 nmol/day)**	37.2 ± 0.2	36.7 ± 0.1	36.4 ± 0.2 *	36.5 ± 0.2 *
**Table 6B Changes in T_IBAT_ following SC infusions of OT or vehicle in 4-h fasted** **female DIO DBA/2J mice**				
**SC**	**Week 1**	**Week 2**	**Week 3**	**Week4**
	**Temp (°C)**	**Temp (°C)**	**Temp (°C)**	**Temp (°C)**
**Vehicle**	37.3 ± 0.2	37.1 ± 0.2	37.2 ± 0.1	37.3 ± 0.1
**OT (16 nmol/day)**	37.1 ± 0.1	36.8 ± 0.2	36.6 ± 0.1 *	36.6 ± 0.2 *
**OT (50 nmol/day)**	37.2 ± 0.2	36.9 ± 0.2	36.7 ± 0.2 *	36.6 ± 0.2 *

**Table 7:** Plasma measurements following chronic systemic infusions of OT (16 and 50 nmol/day) or vehicle in female HFD-fed DBA/2J mice. Data are expressed as mean ± SEM. Different letters denote significant differences between treatments. Shared letters are not significantly different from one another (N = 7/group).

SC Treatment	Vehicle	OT (16 nmol/day)	OT (50 nmol/day)
**Leptin (ng/mL)**	5.6 ± 1.1 ^a^	7.2 ± 1.1 ^a^	8.6 ± 1.3 ^a^
**Insulin (ng/mL)**	6.3 ± 1.1 ^a^	8.1 ± 0.4 ^a^	9.5 ± 2.2 ^a^
**Glucagon (pmol/L)**	12.2 ± 2.2 ^a^	21.0 ± 2.2 ^b^	14.2 ± 3.3 ^ab^
**FGF-21 (pg/mL)**	1158.6 ± 112 ^a^	1505.2 ± 174.1 ^a^	1577.1 ± 131.6 ^a^
**Irisin (mg/mL)**	3.7 ± 0.2 ^a^	4.0 ± 0.3 ^a^	3.8 ± 0.5 ^a^
**Adiponectin (mg/mL)**	8.6 ± 0.4 ^a^	9.5 ± 0.5 ^a^	8.1 ± 0.6 ^a^
**Blood Glucose (mg/dL)**	159.58 ± 5.7 ^a^	153.6 ± 5.2 ^a^	151.4 ± 4.0 ^a^
**FFA (mEq/L)**	0.2 ± 0.02 ^a^	0.2 ± 0.02 ^a^	0.2 ± 0.03 ^a^
**Total Cholesterol (mg/dL)**	98.5 ± 2.8 ^a^	112.4 ± 4.0 ^a^	112.0 ± 6.7 ^a^

## Data Availability

All relevant data is contained within the article: The original contributions presented in the study are included in the article. Further inquiries can be directed to the corresponding author.
